# Transcriptome profiling of two *Moringa* species and insights into their antihyperglycemic activity

**DOI:** 10.1186/s12870-022-03938-6

**Published:** 2022-12-02

**Authors:** K. Mohamed Shafi, Radha Sivarajan Sajeevan, Sania Kouser, Chethala N Vishnuprasad, Ramanathan Sowdhamini

**Affiliations:** 1 National Centre for Biological Sciences (TIFR), GKVK campus, Bangalore, India; 2 Centre for Ayurveda Biology and Holistic Nutrition, The University of Trans-Disciplinary Health Sciences & Technology (TDU), Yelahanka, Bangalore, 560064 Karnataka India; 3 Swedish University of Agricultural Sciences, SE-23422 Lomma, Sweden; 4 Institute of Bioinformatics and Applied Biotechnology, Bangalore, 560100 India

**Keywords:** Moringaceae, Diabetes, RNA-seq, Transcript expression, Secondary metabolites, Enzyme inhibition

## Abstract

**Background:**

*Moringa concanensis* Nimmo (MC), a plant that resembles *Moringa oleifera* Lam. (MO), has less scientific information but has traditionally been used as a medicinal plant. *Moringa* species have long been known for their medicinal qualities, which include antioxidant, anti-inflammatory, anticancer, and antihyperglycemic effects. We investigated the antidiabetic potential of MC and MO species in this study by using transcriptome profiling, metabolite analysis, and *in vitro* assay studies.

**Results:**

Our transcriptome analysis revealed the expression of enzymes involved in the biosynthesis of quercetin, chlorogenic acid, and benzylamine, all of which have previously been shown to have antidiabetic activity. We compared the expression patterns of five different tissues from MC and MO and it was found that the key enzymes involved in the biosynthesis of these compounds were highly expressed in leaf tissue. The expression estimated by MC transcriptome data in different tissues was verified using RT-qPCR analysis. The amount of these compounds was further quantified in the crude leaf extract of both species and found that MC had a higher abundance of quercetin and chlorogenic acid than MO. The crude leaf extract from both MC and MO were further tested *in vitro*, and the results demonstrated strong inhibitory activity for α-glucosidase and DPP-IV enzymes. Our findings suggest that compounds in leaf tissue, such as quercetin, benzylamine, and chlorogenic acid, could play a significant role in this antidiabetic activity. In addition, when comparing MO plants, we found that MC had a slightly higher effect in expression, abundance, and inhibitory activity.

**Conclusions:**

This study presents the first report of MC transcriptome data, as well as a comparison of its anti-diabetic activity to MO. Our analysis discussed the significance of leaf tissue in antidiabetic activity compared to other tissues of both species. Overall, this study not only provides transcriptome resources for *Moringa* species, but also sheds light on antidiabetic potential of both species.

**Supplementary Information:**

The online version contains supplementary material available at 10.1186/s12870-022-03938-6.

## Background

The family Moringaceae has a single genus *Moringa*, with 13 species, of which two species have been recorded from India, *Moringa oleifera* Lam. (MO) and *Moringa concanensis* Nimmo (MC) [1]. MO is a plant of great interest because of its numerous health benefits, including anti-inflammatory, antioxidant, antimicrobial, anti-trypanosomal, antihyperglycemic, anticancer, and antihypertensive activity [2]. This species has received wide attention and is now being cultivated worldwide. MC, on the other hand, is a tree that highly resembles MO and is found in the Konkan region of India. Despite its traditional use as a medicinal plant, very little scientific information is currently available at the transcriptome and metabolome levels. It has demonstrated great potential and diversity as a medicinal plant and deserves further investigation [3]. MC has traditionally been used for various purposes [4], and because the plant closely resembles MO, these plants may share similar medicinal properties; however, secondary metabolites for medicinal activities have yet to be investigated in the case of MC.

The current study focuses on antidiabetic activity, one of the many reputed health benefits of *Moringa* plant. Diabetes is a severe chronic metabolic disorder that has sparked worldwide interest. This disease ranks seventh in terms of mortality rate and has emerged as a significant threat to human health [5]. Plants have been used as dietary supplements and traditional treatment regimens for a variety of diseases for hundreds of years all over the world. Natural products may prove to be a more effective treatment for diabetes with fewer side effects than currently available molecules [6]. A large number of traditionally claimed plant medicines have been tested for diabetes so far, with some showing promising therapeutic potential. MO is one of those plants that is also used for various biological activities. Several studies have shown that the leaves of *Moringa* have antidiabetic properties [7, 8].

Many classes of secondary metabolites, such as alkaloids, phenolics, and flavonoids, have shown promising antidiabetic activities. MO is highly rich in antioxidants and bioactive plant compounds. The three well-known phytochemicals found in this plant that have antidiabetic activity are quercetin, chlorogenic acid, and benzylamine [9]. MO leaves contain a high concentration of quercetin, a potent antioxidant with numerous therapeutic properties. The obese Zucker rat model of metabolic syndrome has demonstrated antidyslipidemic, hypotensive, and antidiabetic effects [10]. Another secondary metabolite, chlorogenic acid, a phenolic acid found in MO leaves, is an ester of dihydrocinnamic acid (caffeic acid) and quinic acid, which has been shown to improve glucose metabolism in the rat liver by inhibiting glucose-6-phosphate translocase and lowering hepatic gluconeogenesis and glycogenolysis [11, 12]. Moringine, an alkaloid isolated from root bark, was later chemically identified as benzylamine, which is also found in the leaves MO. The hypoglycemic effect of plants was thought to be mediated by this substance [13].

Numerous *in vitro* studies have been conducted on antidiabetic activity of MO. However, MC has received little attention in this regard. A recent study [14] reported that the ethanolic extract of MC leaves showed antihyperglycemic activity when tested on glucose, insulin, biochemical, and lipid profile in experimental diabetic rat models induced with streptozotocin (STZ). α-glucosidase, α-amylase, and DPP-IV are considered as important targets for the management of type 2 diabetes. Antidiabetic drugs can reduce glucose absorption in the digestive tract by inhibiting carbohydrate-hydrolysing enzymes such as α-glucosidase and α-amylase. Inhibiting these enzymes causes carbohydrate digestion to be delayed and prolonged, resulting in slower glucose absorption and a lower postprandial plasma glucose rise [15]. DPP-IV is a serine protease found on cell surfaces. This enzyme is responsible for the rapid degradation of incretins such as GLP-1 and GIP. Inhibiting DPP-IV prolongs the action of incretins, which stimulate insulin secretion. Hence, inhibition of DPP-IV is used as a therapeutic strategy in treating Type 2 diabetes [16].

The use of certain plant parts in traditional medicine can be explained scientifically by using transcriptome data and chemical identification using mass spectrometry. A Comparative investigation following the computational research and *in vitro* analyses will reveal the commonalities and differences between plant activities. In our previous study, we identified candidate genes involved in biosynthesis and selected secondary metabolites, vitamins, and ion transporters from MO. The transcriptome analysis revealed the abundance of the enzyme expression in different plant tissues [17]. In this study, we present the first report of transcriptome assembly and analysis of MC, as well as a comparison of antidiabetic activity with MO. Three secondary metabolites were chosen based on their reported antidiabetic properties in these plants, and the enzymes involved in the biosynthesis pathway were explored. In addition, *in vitro* experiments were carried out to investigate the potential antidiabetic activity of MC and MO.

## Results

**Fig. 1:**
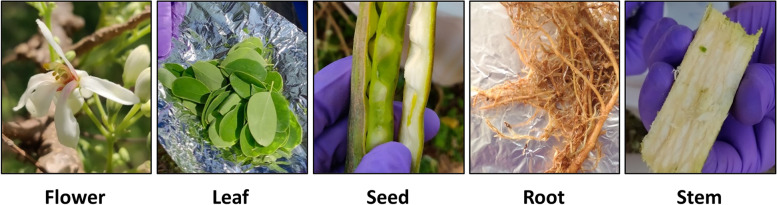
Morphology of MC plant. Five different tissues used for transcriptome sequencing

The antidiabetic potential of MO and MC plants was investigated using transcriptome profiling, metabolites quantification, and *in vitro* assay studies. Five different tissues from MC were used for sequencing **(**Fig. 1**)**, while MO RNA-seq reads were utilized from our previous study. The abundance of transcripts in both plants was compared across tissues. Secondary metabolites with antidiabetic potential were quantified from both plants, and inhibitory activity for the enzymes α-amylase, α-glucosidase, and DPP-IV was tested.

### Transcriptome sequencing and assembly

A total of 15 cDNA libraries were constructed from five different tissues (flower, leaf, seed, root, and stem) of MC species. These libraries were sequenced in duplicates using the Illumina HiSeq 2500 platform and generated around 705 million reads with an average length of 100 bp. High-quality clean reads were chosen for the assembly after removing ambiguous reads identified by FASTQC (Additional File 1). We used MO genome as a reference for the transcriptome assembly because the reads from MC could map to the genome with an alignment rate of 91%. Trinity was used to perform a combined assembly for five different MC tissues. MC Reads were first aligned to the MO genome and the alignment was used for the transcriptome assembly. The abundance of each transcript was estimated and the transcripts belonging to TPM values equal to or more than one was considered for further analysis. The final assembly yielded 114097 transcripts with a total length of 157.9 Mb and with an N50 of 2635 bp (Table 1). This assembly revealed 40.36% GC content. Separately, we obtained clean RNA-seq reads of MO (PRJNA394193) from our previous study [17] and assembled them using the same protocol. About 270 million reads were assembled to 63103 transcripts with an N50 of 2066 bp. The assemblies were further tested for completeness using BUSCO against Viridiplantae, Eudicotes, and Embryophytae databases. MC had greater than 95% completeness against all three databases. In the case of MO assembly, BUSCO obtained more than 80% completeness against these databases (Additional File 2).

**Table 1:** Transcriptome assembly statistics of MC and MO

	***Moringa concanensis* (MC)**	***Moringa oleifera* (MO)**
**Transcripts**		
Number of sequences	114097	63103
Total length	157966670 bp	75486012 bp
Longest sequence	16976 bp	10561 bp
Shortest sequence	197 bp	201 bp
N50	2635 bp	2066 bp
GC content	40.36%	41.74%
**Unigenes**		
Number of sequences	42245	32048
Total length	17184777 bp	11533090 bp
Longest sequence	5103 bp	2303 bp
Shortest sequence	86 bp	85 bp

**Fig. 2:**
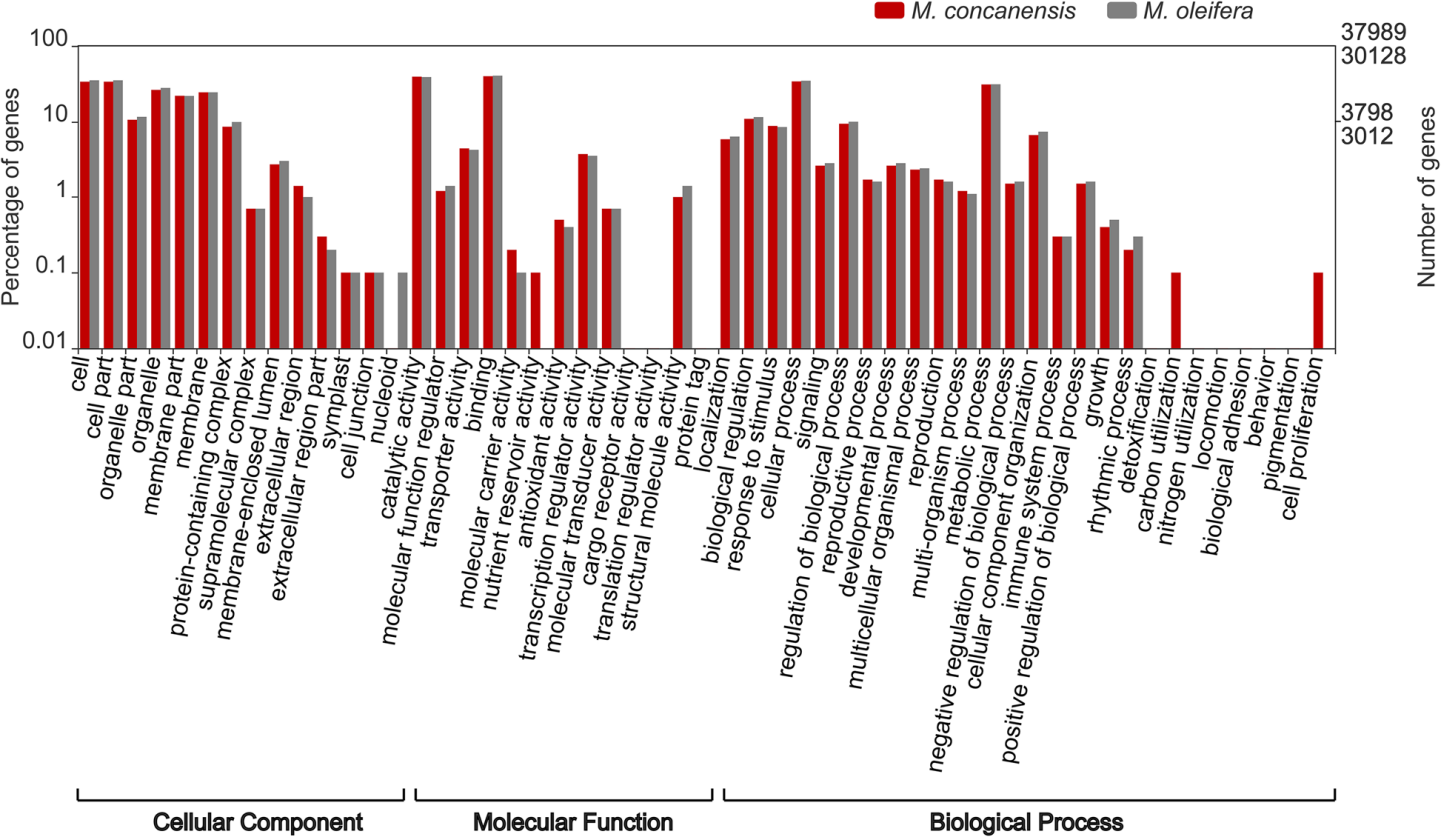
GO enrichment analysis of MC and MO transcriptomes. The graph depicts the overrepresented GO terms in three categories: cellular component, molecular function, and biological process. *x*-axis shows the GO terms and *y*-axis represents the percentage of transcripts in log (10) scale and the number of transcripts from MO and MC

TransDecoder was used to predict coding sequences (unigenes) from both assemblies with a length threshold of 100 amino acid residues. The MC transcriptome assembly predicted 42245 unigenes with an average sequence length of 407 amino acids. Around 94% of unigenes were annotated using BLASTP against the Viridiplantae database (Additional File 3) and HMMSCAN against the Pfam database predicted domains for 87% of the unigenes. MO transcriptome predicted 32048 unigenes and both BLASTP and HMMSCAN were able to annotate 94 and 87%, respectively. Most of the homologues were found from *Theobroma cacao*, a closely related plant, followed by *Cephalotus follicularis* (pitcher plant), *Manihot esculenta* (cassava), *Fagus sylvatica* (beechnut), and *Juglans regia* (walnut) (Additional File 4A). *Cacao* and *Moringa* are members of the taxonomic clade Malvids, while the other species are members of the distantly related clade Fabids. Both MC and MO had an abundance of ‘protein kinase domain’ encoding transcripts (Additional File 4B). They were also significantly enriched in ‘F-box’ and ‘ubiquitin’ related transcripts. Functional enrichment analysis was performed using the GO terms obtained from the homologous sequences (Fig. 2). We observed that the ‘metabolic’ and ‘cellular processes’ were overrepresented in the ‘biological process category’. Also, binding and catalytic activity-related terms were highly enriched in the molecular function category. The functional domains of these unigenes were further annotated using HMMSCAN against Pfam database. Pfam domains were predicted for 87% of the sequences from both species. ‘PPR’ and ‘Pkinase’ related domains were abundant in them (Additional File 4C).

### Transcript expression and functional annotation

The assembled transcripts were mapped back to the reads in order to determine the abundance of each transcript. RSEM analysis was used to estimate TPM values, which were then compared between tissues. We analysed top ten expressed transcripts in different tissues of both species. Most of these transcripts were either uncharacterized or lacked a functional domain (Additional File 5). Transcripts that were highly expressed in different tissues include ‘ubiquitin related’ and ‘elongation factor 1-alpha’. One of the common transcripts that are highly expressed in the flower tissue of both species was ‘pectin methylesterase inhibitor’. This gene is involved in plant immune response during stress conditions. In MO leaf tissue, the ‘glucan endo-1,3-beta-glucosidase’ transcript was found to be highly expressed. This protein plays an important role in plant pathogen defense. During biotic stress, the amount of this transcript will significantly increase and play a major role in defense response. ‘Germin-like proteins’ are glycoproteins involved in plant responses to various abiotic and biotic stresses, particularly pathogens. These transcripts, like other transcripts involved in the defense response, were found to be the most abundant in MO leaf tissue. Interestingly, many ‘Annexin’ proteins were abundant in MO seed tissue. These calcium binding proteins serve a variety of functions in plants. Instead of ‘Annexin’, ‘Laccase enzyme’ was found in the top ten in MC. This is a multicopper protein found in higher plants and fungi. The most abundant transcript in MC root tissue was a proline-rich protein, which is important for plant development. ‘Myrosinase’ and ‘Beta-glucosidase ‘were also found to be highly expressed. These genes primarily function in crop protection and defense response.

### Gene family analysis with closely related species

**Fig. 3:**
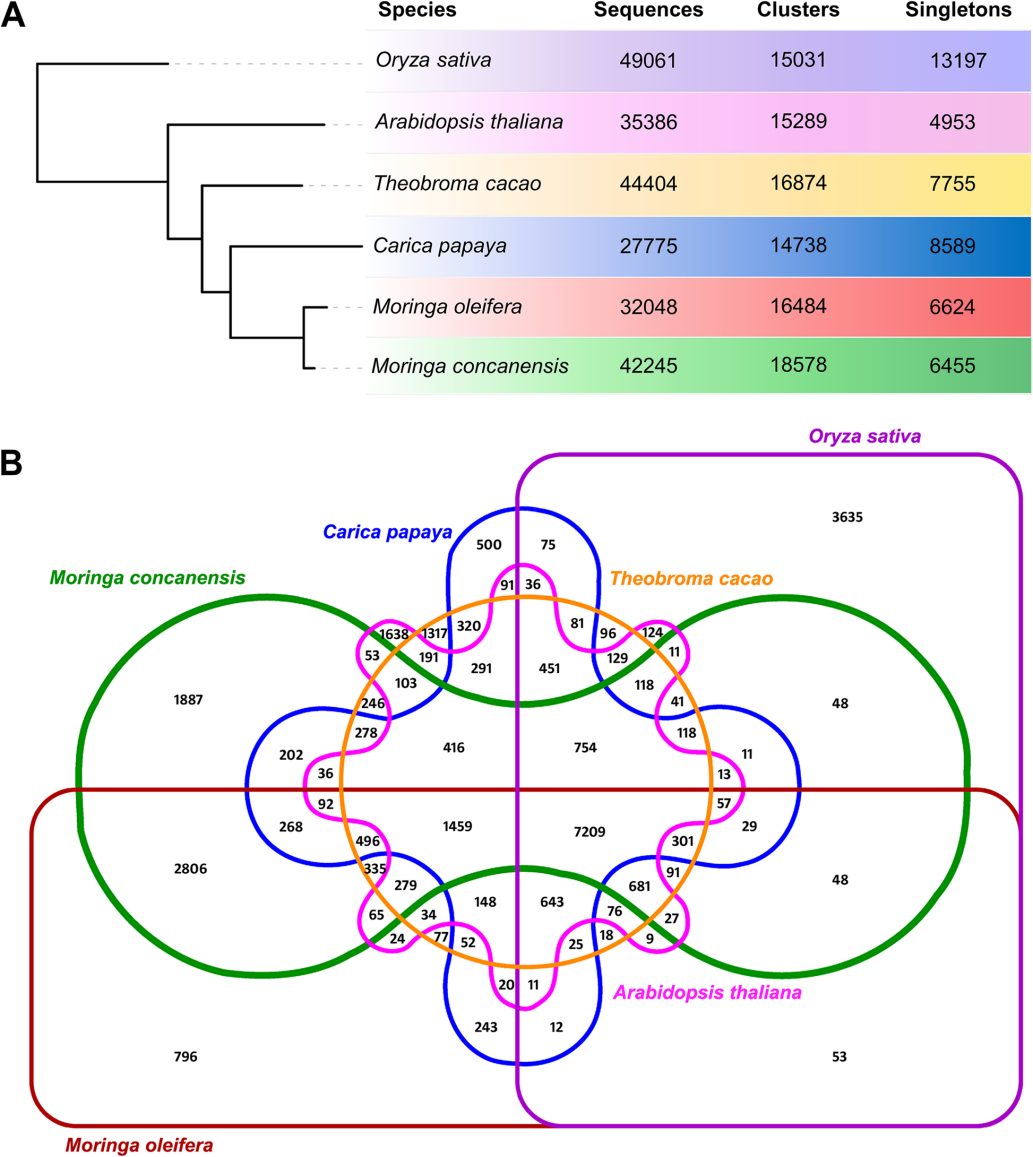
Gene family analysis of *Moringa* species with closely related plants. **A** The phylogeny tree was generated using species evolutionary distance. Total number of sequences, clusters, and singletons identified from the analysis has been provided. **B** A venn diagram showing the number of orthologs clusters shared among six species studied

Gene families from *Moringa* species and closely related plants were compared further. In addition to close evolutionary plants like *T. cacao* and *Carica papaya*, we chose model plants such as *Arabidopsis thaliana* and *Oryza sativa* for this study (Fig. 3A**)**. Similar gene families among these species were clustered using OrthoVenn2. In total, 389125 sequences from six species were used as input, resulting in 29794 orthologous clusters and 802 single-copy gene clusters. There were 76744 genes in 7209 clusters with all species present. The clusters formed between the species are depicted by a Venn diagram (Fig. 3B). The functions of these cluster groups were further investigated. The GO terms of the genes in each cluster were used for enrichment analysis. MC and MO had 6455 and 6624 unigenes, respectively, that were unique (singletons) to them. MO singletons were enriched for ‘chlorophyll metabolic process’, ‘ozone response’, and ‘protein glycosylation’ as biological processes. In contrast, MC singletons were enriched for ‘unidimensional cell growth’, ‘sodium ion import across the plasma membrane’, and ‘polysaccharide catabolic process’. The gene families that were shared by all species showed an enrichment of biological processes involving ‘RNA modification translation’, ‘defense response’, and ‘transcription regulation’. The most enriched term in terms of molecular function was protein ‘serine/threonine kinase activity’, followed by ‘DNA binding’.

Separately, we investigated the MC and MO gene families and identified 14627 clusters, of which 46601 were shared by both. Over 80% of MC sequences predicted MO homologues, and about 75% of MO sequences also found MC homologues. This implies that the genetic make-up of these two species is also quite similar. We noticed many of the gene families shared by these species were transcription factors (TFs). TFs play an important role in the regulation of gene families. We predicted TFs from all six species and classified them into different TF families. In our analysis, C2H2, WD40-like, MYB-HB-like, bHLH, and PHD were the most abundant transcription factors (Additional File 6). These transcription factors were present in nearly identical amounts in MC, MO, and other closely related species. We noticed that the number of transcription factors in MC was more similar to *T. cacao*. MO, on the other hand, possessed fewer transcription factors and was more closely related to *C. papaya*.

### Expression of the enzymes involved in the biosynthesis pathway of metabolites

*Moringa* species contain a variety of phytoconstituents, including alkaloids, phenolic acids, glucosinolates, flavonoids, saponins, tannins, and steroids. Some of the secondary metabolites, quercetin, benzylamine, and chlorogenic acid, are highly abundant in MO leaves and widely studied for their antidiabetic potential. We analysed the expression of the enzymes involved in the biosynthesis of these three metabolites from MC and MO using a combination of computational analysis and experimental validation.

### Quercetin biosynthesis

**Fig. 4:**
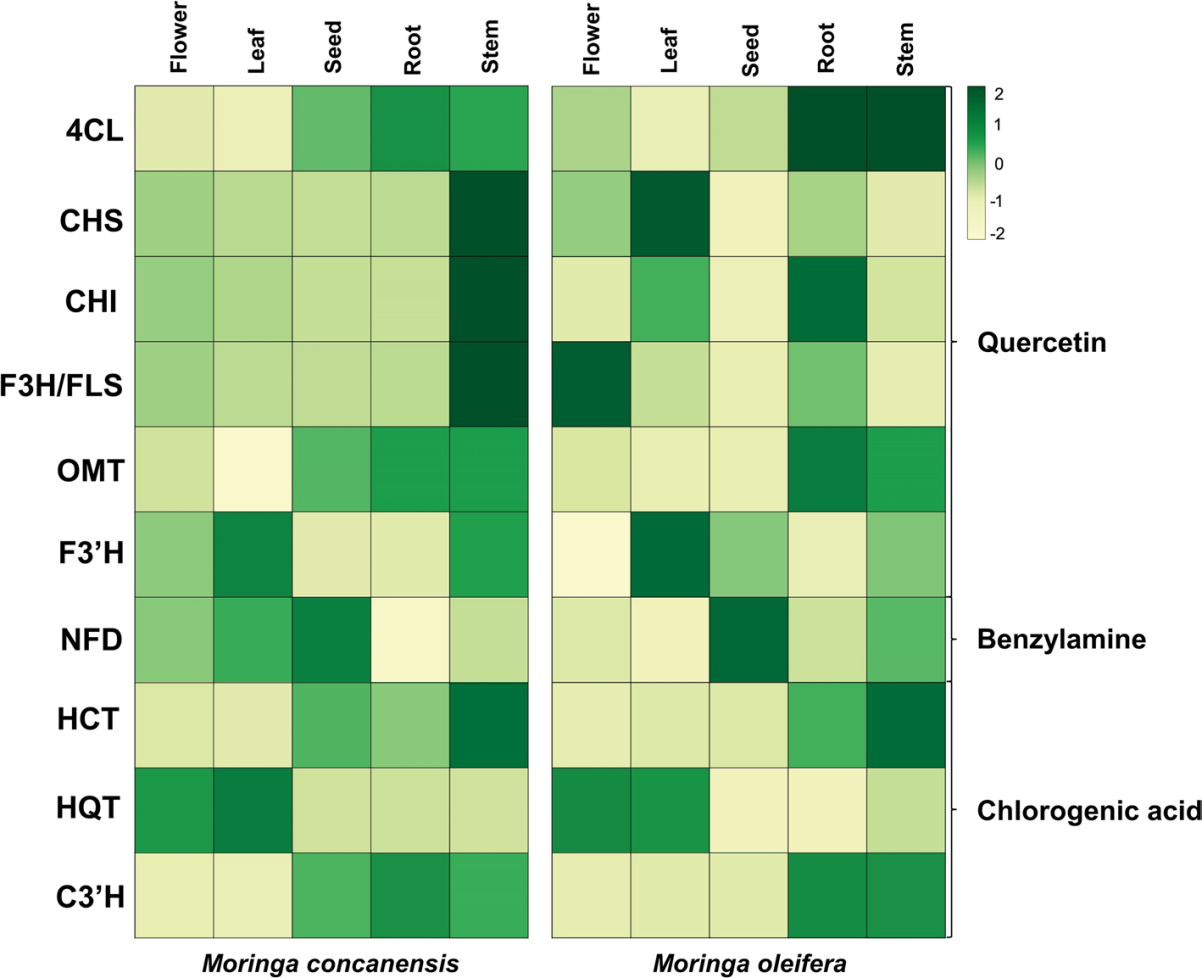
Transcript expression of enzymes in five different tissues of MC and MO. The heatmap showing expression (TPM in log (10) values) of candidate transcripts involved in the biosynthesis of quercetin, benzylamine, and chlorogenic acid in five different tissues

**Fig. 5:**
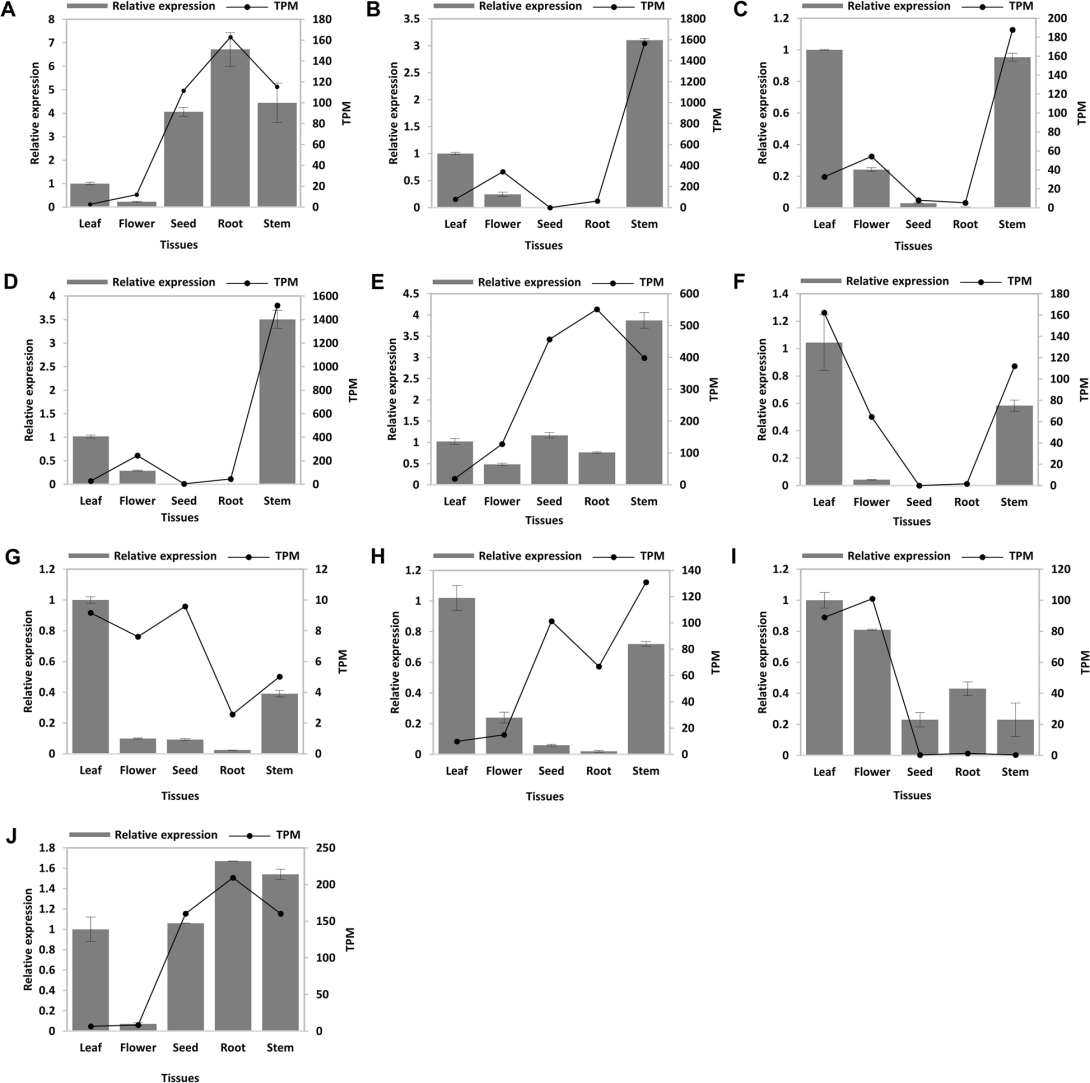
Validation of transcript expression using RT-qPCR analysis. The enzymes involved in the biosynthesis of quercetin, benzylamine, and chlorogenic acid (**A**) 4CL, (**B**) CHS, (**C**) CHI, (**D**) F3H/FLS, (**E**) OMT, (**F**) F3’H, (**G**) NFD, (**H**) HCT, (**I**) HQT and (**J**) C3’H were quantified in five different tissues of MC using RT-qPCR. The grey-scale bars represent relative enzyme coding transcript expression in flower, leaf, seed, root, and stem by RT-qPCR analysis (left *y*-axis). The data represents mean values of three biological and three technical replicates (total 9 replicates for each sample). Black line represents TPM values of the transcripts in flower, leaf, seed, root, and stem by RNAseq (right *y*-axis). The error bars represent the standard error between replicates in RT-qPCR analysis

Quercetin is a flavonoid, a potent antioxidant with a variety of therapeutic properties. It is commonly found in many vegetable and fruit species. Several studies have reported quercetin to be effective in lowering blood glucose levels, and it is highly abundant in MO leaves. The enzyme involved in quercetin biosynthesis was investigated. Seven enzymes are involved in the biosynthesis of quercetin from 4-coumarate. The transcripts encoding the first enzyme coumaroyl CoA ligase (4CL) were identified from the MC and MO transcriptomes using homologue sequences. A combination of phylogeny and FIR mapping analysis helped us to ascertain the true hits (Additional File 7). We observed that 4CL (EC: 6.2.1.12) was expressed in seed, root, and stem tissues of MC compared to other tissues, whereas in MO, it was highly expressed in stem tissue only (Fig. 4, Additional File 8). Other enzymes involved in the pathway, chalcone synthase (CHS), chalcone flavanone isomerase (CHI), flavonol 3 hydroxylase (F3H), flavonol synthase (FLS), tricin synthase (OMT), and flavonoid 3 monooxygenase (F3’H) were studied further. The abundance of these enzymes was analysed in different tissues of both plants and we noticed CHS (EC: 2.3.1.74), CHI (EC: 5.5.1.6), and a bifunctional enzymes F3H (EC:1.14.20.6) / FLS (EC: 1.14.11.9) were highly expressed in stem tissue of MC, whereas in MO, these were expressed in leaf, root, and flower respectively. The expression levels of OMT (EC: 2.1.1.175) were high in root and stem compared to other tissues in both plants. The key final enzyme in the production of quercetin, F3’H (EC: 1.14.14.82), is a cytochrome p450 family enzyme. The transcript encoding this enzyme from MC (Mcon_489_c3618_g1_i1) and MO (Mole_14295_c0_g1_i1) were seen highly expressed in leaves. The expression of these seven enzymes in different tissues of MC was further validated using RT-qPCR analysis (Fig. 5A-F).

### Benzylamine biosynthesis

Benzylamine (Moringine), an alkaloid compound isolated from MO, has been shown to have antidiabetic properties in several *in vivo* studies. This compound is produced in plants by the enzyme N-substituted formamide deformylase (NFD) from N-benzylformamide, and in bacteria by NFD on the reactant N-benzylcyanide. NFD enzyme (EC: 3.5.1.91) belongs to the amidohydrolase superfamily. We used bacteria NFD sequences as a query for searching the enzyme in the MC and MO transcriptome. Through our transcriptome analysis and sequence searches, we identified hits belonging to the NFD enzyme from both plants. Phylogeny analysis and FIR mapping were carried out with the homologue sequences to ascertain the hits from the transcriptome (Additional File 7). In both bacterial and plant NFDs, four metal-binding residues are conserved. We observed the transcript Mcon_1382_c4_g1_i1 from MC was found to be highly expressed in seed and leaf, whereas Mole_14496_c0_g1_i3 from MO was found to be mostly expressed in seed and stem compared to other tissues (Fig. 4, Additional File 8). Despite the fact that the TPM values estimated for this enzyme in different tissues were lower, our findings matched previous benzylamine observations in MO. The presence of benzylamine in root and leaf tissues has previously been reported. RT-qPCR analysis was used to confirm the expression of the NFD enzyme in MC (Fig. 5G).

### Chlorogenic acid biosynthesis

Chlorogenic acid is a phenolic compound from the hydroxycinnamic acid family which is found in many vegetables, fruit, coffee plants, etc. The metabolite is derived from phenylalanine and conversion to p-Coumaroyl-CoA is the committed step in the phenylpropanoid pathway. Its chemical structure consists of a caffeic acid moiety and a quinic acid moiety. This compound has demonstrated its ability to lower the blood sugar level in mice models. Chlorogenic acid pathway includes three major enzymes which synthesize chlorogenic acid with different substrate specificities, Hydroxycinnamoyl-CoA shikimate hydroxycinnamoyl-transferase (HCT), Hydroxycinnamoyl-CoA quinate hydroxycinnamoyl-transferase (HQT) and p-Coumaroyl ester 3’-hydroxylase (C3’H) (Additional File 7). HCT (EC: 2.3.1.133) and HQT (EC: 2.3.1.99), two similar enzymes from the acyltransferase family, yielded multiple hits from both MC and MO. We performed phylogeny analysis and FIR mapping with homologues sequences to identify the transcript encoding enzyme. Both enzymes formed a distinct cluster in the phylogeny. These enzymes have conserved two BAHD catalytic motifs, HXXXDG and DFGWG. The differences in conserved residues such as H153, and H154 in HCT in the catalytic motif HXXXDG, whereas H153 and T154 in HQT helped us classify the sequences into two groups. Our transcriptome analysis showed a high abundance of HCT enzymes in the root and stem compared to other tissues for both plants (Fig. 4, Additional File 8). For MC, this enzyme showed expression in seed also. Based on previous studies, HQT is considered one of the key enzymes in chlorogenic acid biosynthesis. This enzyme from both plants (Mcon_489_c129_g1_i2 and Mole_13679_c0_g1_i1) was found to be highly expressed in leaf tissue, followed by flower tissue. The third enzyme, C3'H (EC: 1.14.13.36), a cytochrome P450 enzyme was observed for both plants with a similar expression pattern to the HCT enzyme. The MC transcriptome expression pattern was supported by RT-qPCR analysis (Fig. 5H-J).

### Quantification of metabolites in leaf tissue of *Moringa* species

Our investigation into the transcriptome of MC and MO led to additional research on leaf tissue. A crude extract of leaf tissue from both plants was prepared using a combination of solvent mixture methanol (70%) and water (30%). The secondary metabolites quercetin, benzylamine, and chlorogenic acid were quantified using HPLC analysis assisted by standard compounds (Additional File 9). The quercetin compound was found to be 30 times more abundant in MC than in MO (Additional File 10A). Chlorogenic acid was also found to be more abundant in MC than in MO. Benzylamine levels in leaf tissue of both plants were nearly identical. LC-MS profiling was performed to detect other compounds in the crude extract of leaf tissue. In both plants, we find many similar compounds from different classes (Additional File 10B). The antidiabetic activity of the leaf tissue may be due to a combination of quercetin, benzylamine, and chlorogenic acid, as well as other compounds found in the tissue.

### *In vitro* enzyme inhibition assays

The MC and MO leaf crude extracts were tested *in vitro* for their antidiabetic potential. The carbohydrate metabolizing enzymes α-glucosidase and α-amylase are important pharmacological targets for type 2 diabetes management. Postprandial blood sugar levels can be controlled by inhibiting these enzymes. Both MC and MO crude extracts of leaf tissue were found to inhibit α-glucosidase and α-amylase in a concentration-dependent manner (Table 2). For α-glucosidase activity, MC showed better inhibitory activity than MO at a lower concentration. At 1.56 mg/ml concentration, MC showed 92.98 ± 4.08% inhibition, whereas MO showed 22.14 ± 2.48% inhibition only. MO showed 88.75 ± 5.50% activity in almost four times higher concentration (6.25 mg/ml). Both MC and MO calculated an IC_50_ of 0.73 and 3.53 mg/ml, respectively. MO exhibited a maximum of 76.90 ± 10.97% inhibition for α-amylase, while MC inhibited at a maximum of 78.84 ± 9.36% at the highest concentration of 50 mg/ml. At a lower concentration of 25 mg/ml, MO showed 70% inhibition, while MC activity was substantially lower. The IC_50_ was determined to be 17.94 and 29.18 mg/ml for MO and MC, respectively. At lower concentrations, MO inhibited α-amylase more effectively than MC.

**Table 2:** α-glucosidase, α-amylase, and DPP-IV inhibitory activity for crude leaf extract of MC and MO. Percentage of inhibition showed in Mean ± SD of three replicate values

**Enzyme**	**Concentration (mg/ml)**	**Percent Inhibition**			
		**MO**	**IC**_**50**_	**MC**	**IC**_**50**_
α-glucosidase	12.5	100 ± 0.48	3.53 mg/ml	99.29 ± 1.20	0.73 mg/ml
	6.25	88.75 ± 5.50		98.94 ± 1.38	
	3.12	47.38 ± 3.03		98.85 ± 0.41	
	1.56	22.14 ± 2.48		92.98 ± 4.08	
	0.78	10.89 ± 1.79		55.60 ± 22.93	
	0.39	6.18 ± 0.91		10.05 ± 9.27	
α-amylase	50	76.90 ± 9.36	17.94 mg/ml	78.84 ± 10.97	29.18 mg/ml
	25	65.12 ± 11.95		11.62 ± 5.49	
DPP-IV	12.5	93.80 ± 1.91	1.50 mg/ml	91.82 ± 2.65	1.09 mg/ml
	6.25	85.39 ± 4.33		83.91 ± 3.07	
	3.12	75.22 ± 7.44		69.97 ± 5.54	

Dipeptidyl peptidase-IV (DPP-IV) inhibition was also tested on the crude extract. This enzyme is important in maintaining normal blood sugar levels in the body. Incretins secreted in response to meal consumption increase insulin release into the blood, and DPP-IV rapidly deactivates and inhibits their activity. In the case of diabetes, inhibiting this enzyme increases incretin activity and insulin production, leading to improved glucose control. The inhibition of DPP-IV by leaf crude extracts of MC and MO was tested using a fluorometric assay in dose dependent manner (Table 2). The assay demonstrated strong inhibition for both MC (91.82 ± 2.64%) and MO (93.8 ± 1.9%) at a maximum concentration of 12.5 mg/ml. Even at the lowest assay concentration (3.125 mg/ml), MC and MO showed an inhibition of 69.97 ± 7.45 and 75.22 ± 5.54%, respectively. When compared, MC and MO had similar inhibitory activity, with IC_50_ values of 1.50 and 1.09 mg/ml, respectively.

## Discussions

### Antidiabetic property of *Moringa* species

MO has been used as a food source in traditional medicine because of its wide range of medicinal and nutritional activities. These characteristics are primarily due to the abundance of various phytochemicals present in different parts of this plant [18]. Various parts of the MO plant are being researched for their potential benefits in the treatment of metabolic conditions, particularly diabetes. Metabolic disorders affect a large percentage of the population and mostly lead to chronic complications. The current rise in the prevalence of diabetes and other metabolic disorders necessitates improved lifestyle and nutritional strategies, as well as the development of more effective therapeutic options [19]. MO plant has been shown to have antihyperglycemic activity in animal models [20]. Genome and transcriptome studies for MO have already been conducted [17, 21]. In this study, we discuss the antihyperglycemic activity of MC, another member of the Moringaceae family found in India that closely resembles MO. This plant has traditionally been used as a medicinal plant, but there is little scientific information available so far. In STZ-induced diabetic rats, this plant was studied for its potential antidiabetic mechanism [14]. Compared to MO, relatively little research has been done on the closely related species MC. We investigated the antidiabetic potential of both MC and MO through transcriptome profiling, metabolite analysis, and *in vitro* assay studies.

### Comparative analysis between transcriptome of MC and MO

Despite the similar morphology of MC and MO, we anticipate that these two species will have similar medicinal properties. To explore the antidiabetic potential of both plants, we carried out transcriptome profiling for five different tissues. For MC sequencing, new samples were obtained, and for MO, previous transcriptome data were used [22]. Both data assembled 114097 (42245 unigenes) and 63103 (32048 unigenes) transcripts for MC and MO, respectively. The abundance of each transcript in different tissues of both plants showed transcripts that have highly expressed. We observed many transcripts that are highly expressed in various tissues and are mostly involved in ‘ubiquitination’, ‘defense response’, and various developmental processes. The transcriptomes of both species were functionally annotated, and over 90% of unigenes were found to have homologues in the Viridiplantae database. The enrichment analysis of these unigenes showed an abundance of GO terms related to ‘metabolic and cellular’ processes. We also observed GO terms associated with binding and ‘catalytic activity’ were significantly overrepresented in both MC and MO. We further noticed that the majority of the homologous sequences for both species were predicted from the evolutionary close plant *T. cacao*. So, we chose two evolutionary close plants *T. cacao* and *C. papaya*, as well as two model plants *A. thaliana* and *O. sativa* for a gene family analysis with MC and MO. The sequences from all six species were grouped into 29794 orthologous groups, with 7209 (76744 genes) shared by all species. These genes were mainly enriched with GO terms like ‘RNA modification translation’, ‘defense response’, ‘transcription regulation’, and ‘DNA binding’. MC and MO had 6455 and 6624 singleton genes that did not cluster with other species, respectively. MO singletons were enriched in ‘chlorophyll metabolism’, ‘ozone response’, and ‘protein glycosylation’, whereas MC singletons were enriched for ‘unidimensional cell growth’, ‘sodium ion import across the plasma membrane’, and ‘polysaccharide catabolic processes’. We noticed that MO and MC shared approximately 80% sequence similarity, indicating that they are genetically related. Many of their gene families shared between these species were TFs. They are crucial in gene regulation. The most abundant TFs in both species were C2H2, WD40-like, MYB-HB-like, bHLH, and PHD. The enrichment of these TFs was nearly equal when compared between MC and MO as well as to closely related species.

### The secondary metabolites involved in the antidiabetic activity

MO leaves are the most widely researched and they have been shown to be beneficial in several chronic conditions, including hypercholesterolemia, high blood pressure, diabetes, cancer, and overall inflammation. Three important metabolites that are found in leaf tissue and are known for their antidiabetic activity are quercetin, chlorogenic acid, and benzylamine [9]. The expression of enzymes involved in the biosynthesis of these compounds was assessed in different tissues of MC and MO. Compared to other tissues, we noticed that the final enzymes in each pathway were abundant in the leaf tissue. Quercetin is a potent antioxidant, abundant in many plants. This substance is well-known for its therapeutic properties, which include an antidiabetic effect [23]. Seven enzymes are involved in the biosynthesis of quercetin, and the majority of them were found to be expressed in stem tissue. The last key enzyme, F3'H, was highly expressed in the leaf tissue of both plants. The second compound, benzylamine, was initially isolated from MO [13]. Hence, this substance is also known as moringine. The antihyperglycemic activity of this compound has previously been reported [24]. The synthesis of benzylamine involves a single enzyme, NFD, which was found to be relatively abundant in leaf and seed tissues. Chlorogenic acid, the last compound we looked at, is a substance that can be found in a wide variety of fruits and vegetables. This compound has been claimed to modulate glucose and lipid metabolism [25]. The biosynthesis of chlorogenic acid involves three major enzymes. The key enzyme HQT was found to be highly expressed in the leaf and flower, while HCT and C3'H were found in the root and stem. Using RT-qPCR analysis, we were able to confirm the majority of the expression pattern estimated by using transcriptome studies. Since we identified a high abundance of key enzymes in leaf tissue for all three compounds, we quantified them in leaf tissue from both plants. The HPLC analysis showed that MC leaf tissue contained 30 times more quercetin than MO. Similar amounts of benzylamine were found in both plants, and MC showed a higher amount of chlorogenic acid. We also investigated other compounds present in the leaf tissue and detected them by LC-MS profiling. Overall, our findings show that these metabolites are highly expressed and abundant in both plants leaf tissue and have potential antidiabetic properties.

### Inhibition of enzyme activity

The leaf tissues from both plants were further subjected to *in vitro* analysis. The inhibitory activity of MC and MO leaf tissue crude extracts was tested in a concentration-dependent manner. α-glucosidase, α-amylase, and DPP-IV have been used as major enzyme targets for controlling blood sugar. Inhibitors of α-glucosidase and α-amylase can slow down the breakdown of carbohydrates in the small intestine and reduce the postprandial blood glucose in diabetes. DPP-IV inhibitors can improve glucose homeostasis by modulating the incretin effect by inhibiting DPP-IV action on GLP-1 and GIP, two important incretin hormones. Identifying alternative drugs made from medicinal plants that are more potent and have fewer side effects than currently available drugs is important. HPLC and LC-MS analysis confirmed the presence of quercetin, chlorogenic acid, benzylamine, and other compounds in these extracts. In our analysis, MC leaf extract showed better inhibitory activity for α-glucosidase in lower concertation when compared to MO. The IC_50_ for MC was 0.73 mg/ml, while the IC_50_ for MO was 3.53 mg/ml, which was significantly lower. In the case of α-amylase, MO showed better activity at lower concentrations, with an IC_50_ of 17.94 mg/ml versus 29.18 mg/ml for MC. The extracts were further assayed for DPP-IV inhibition, and both extracts inhibited well, with IC_50_ values of 1.50 mg/ml and 1.09 mg/ml for MC and MO, respectively. Leaf tissue from both plants showed strong inhibitory activity against α-glucosidase and α-amylase, as well as DPP-IV, in our assays. This activity could be attributed to the presence of active phytochemicals such as quercetin, chlorogenic acid, and benzylamine. This suggests that leaf tissue could be a promising candidate for a low-risk, effective treatment for postprandial hyperglycemia.

## Conclusions

In this study, we performed transcriptome profiling of MC and MO. Our research focused on the antidiabetic potential of *Moringa* species, one of the many biological activities attributed to these plants. Transcriptome and RT-qPCR analysis reveal the abundance of enzymes involved in the biosynthesis of bioactive compounds in different tissues of *Moringa* species. Our findings suggest that leaf tissue is important for this activity, which prompted us to conduct additional research by quantifying metabolites in leaf tissue and conducting *in vitro* study. Taken together, this study not only provides transcriptome resources for *Moringa* species, but also sheds light on antidiabetic potential of MC and MO.

## Methods

### Plant materials, RNA isolation, and library preparation

Plant samples for MC were collected from Indian Institute of Horticultural Research (Bangalore, India) and authenticated at the FRLHT herbarium (Voucher specimen number: 119975). Tissue samples in triplicate (flower, leaf, seed, root, and stem) were collected and flash-frozen in liquid nitrogen at the site, transported to the laboratory and stored at -80°C until further use. Total RNA was extracted using the SpectrumTM Plant Total RNA Kit (Sigma-Aldrich) using 75-100 mg of collected tissues. RNA integrity and concentration were evaluated on a Bioanalyzer (Agilent, Inc.). For library preparation and sequencing, samples with RNA integrity number (RIN) 6.5 or above were submitted to AgriGenome Labs Pvt. Ltd. (Kochi, India). A total of 15 libraries were prepared, containing three biological samples for each tissue and were sequenced in duplicates.

### Transcriptome sequencing, assembly and annotation

cDNA libraries prepared for MC were sequenced using Illumina HiSeq 2500 platform V4 chemistry to yield 100 bp paired-end reads. The read quality was evaluated using FASTQC (https://www.bioinformatics.babraham.ac.uk/projects/fastqc). High-quality reads were retained for the assembly after removing ambiguous reads. MO transcriptome reads for five tissues (flower, leaf, seed, root, and stem) were obtained from our previous study (https://www.ncbi.nlm.nih.gov/bioproject/PRJNA394193) [22]. Clean reads from MC and MO were assembled independently. The genome of MO [21] was used as a reference for both species, and guided assembly was performed using the Trinity program [26]. A K-mer size 25 was used for the assembly along with other default parameters in Trinity. BUSCO [27] analysis was carried out to determine the completeness of the transcriptome assemblies. TransDecoder was used to predict coding sequences (unigenes) with at least 100 residues in length. Transcripts and unigenes were functionally annotated using BLASTX and BLASTP [28] against the UniProt [29] Viridiplantae database with an E-value threshold of 1e-5. Functional domains were detected using HMMER [30] program against the Pfam database [31] with default parameters. The enriched GO terms were visualized using WEGO tool (https://wego.genomics.cn/) [32].

### Transcript abundance estimation

RNA-seq Expectation-Maximization (RSEM) was used to estimate relative abundances and expected read counts for each transcript [33]. The reads were aligned to the assemblies and estimated using the Bowtie 2 [34] and eXpress [35], respectively. The 'trinity mode' parameter was used to generate a gene count matrix in addition to an isoform count matrix. TPM (Transcripts Per Million) values were compared across the replicates, and the average value for the transcript in each tissue was reported.

### Gene family analysis

Orthology analysis for *Moringa* species was performed with the well-studied model plants *A. thaliana* (dicot) and *O. sativa* (monocot), as well as evolutionary closely related species such as *T. cacao* and *C. papaya* using OrthoVenn2 (https://orthovenn2.bioinfotoolkits.net) [36]. An E-value of 1e-5 and an inflation value of 1.5 threshold were used for the all-against-all protein comparison. The clusters shared between species were visualized using a Venn diagram. Each cluster was annotated, and functional enrichment analysis was performed using GO terms [37]. Transcription factors were predicted using plantTFcat (https://www.zhaolab.org/PlantTFcat/) [38].

### Investigation of enzymes involved in the biosynthesis pathway

Enzymes involved in the biosynthesis of quercetin, chlorogenic acid, and benzylamine were identified from transcriptome using CAPS_protocol [39]. The pathway information was collected from the PlantCyc database [40] and literature. The seed sequences for the enzymes were collected from Uniprot [29]. Clustal Omega [41] was used to align the queries, which were then used as input for two iterations of jumpstart PSI-BLAST [42] against the transcriptomes with an E-value of 1e-5 and inclusion threshold of 1e-5. The hits were filtered using the threshold of query coverage (70%) and percentage identity (40%). To identify true hits, the filtered hits were aligned with query sequences and functionally important residues (obtained from the literature) were mapped following the phylogeny tree construction using MEGA [43]. The sequences were aligned using Muscle module [44] and maximum likelihood was used to generate phylogeny with bootstrap value 1000.

### Real-Time Quantitative Reverse Transcription PCR (RT-qPCR) Verification

Tissue samples (flower, leaf, seed, root, and stem) were collected in three biological replicates from mature field-grown trees at Indian Institute of Horticultural Research (Bangalore, India). Total RNA was isolated from 100 mg tissues of all the above samples using the Spectrum^TM^ Plant Total RNA Kit (Sigma-Aldrich). Total RNA was treated with DNaseI enzyme (1U, MBI Fermentas, USA) following manufactures protocol to remove the presence of genomic DNA. The quality and quantity were analyzed using agarose gel electrophoresis (1.2 %) and nanodrop. A total of 4 μg total RNA was used for the first-strand cDNA preparation in a 20 μl reaction volume using the SuperScript™ III First-Strand Synthesis SuperMix (Thermo Fisher Scientific) following the manufacturer's protocol. In triplicates, the RT-qPCR was performed using a CFX96 RT-qPCR detection system (Bio-Rad, Hercules, CA, USA) in a final reaction volume of 20 μl containing gene-specific forward and reverse primers (10 pmol/μL each, Sigma Aldrich), cDNA (2 μl), and 10 μl of 2× iQ SYBR green super mix (Bio-Rad, California, USA). The reaction conditions were as follows - initial denaturation of 95 °C for 3 min, followed by 35 cycles - denaturation at 95 °C for 15 s, annealing at 58 °C for 20 s, and extension of 72 °C for 20 s. The melt curve analysis was performed and the 2−∆∆Ct method [45] was used to calculate the relative expression levels using glyceraldehyde 3-phosphate dehydrogenase (GAPDH) as the internal reference. All the primer sequences used in this study are given in (Additional File 11).

### Quantification using HPLC analysis

The leaves of MC and MO were washed with tap water and dried for 24 hours at 40 °C. The dried materials were ground into a fine powder using an electric blender and stored in an airtight container for later use. 5 g of dried powdered materials from both plants were extracted on a magnetic stirrer with 50 ml of Methanol:Water (30:70) % (v/v), and the extracts were filtered through filter paper [46, 47]. One ml of the crude leaf extract from both plants was centrifuged for 5 minutes at 14800 rpm, 4 °C. 10 μl of each sample supernatant was injected into the HPLC-PDA system for analysis. The parameters used for the HPLC-PDA setup are given in (Additional File 12).

### LC-MS profiling

LC-MS profiling was performed to detect compounds in crude leaf extracts of MC and MO. Thirty μl each of the crude leaf extracts from both plants was centrifuged for ten minutes at 148000 rpm and the supernatant was transferred to fresh tubes. The supernatants were spiked with ten μl each of reserpine (positive ion mode) and taurocholate-D8 (negative ion mode). Ten μl supernatants were injected to the system after another round of centrifugation. A pool of plant growth hormones, amino acids, neurotransmitters, phenolic compounds, organic acids, glucose, glucose-6-phosphate, reserpine, and Taurocholate-D8 was used as a standard mix and injected ten μl for analysis. The parameters used for the LC-MS setup are detailed in (Additional File 12).

### α-amylase and α-glucosidase inhibition assay

The enzymes α-amylase (*Aspergillus oryzae*; Cat. No. 10065) and α-glucosidase (*Saccharomyces cerevisiae*; Cat. No. G5003) and other fine chemicals were purchased from Sigma-Aldrich (St. Louis, MI). α-amylase and α-glucosidase inhibition for the crude leaf extract of MC and MO was determined using previously established method [48, 49].

### DPP- IV inhibition assay

DPP- IV activity was determined using the DPP- IV drug discovery Kit (BML-AK499 from Enzo Life Sciences, Farmingdale, NY, USA), according to the kit instructions. We performed fluorometric assay based on the cleavage of 7-amino-4-methylcoumarin (AMC) moiety from the C-terminus of the peptide substrate, which increases its fluorescence intensity at 460 nm. The percentage remaining activity in the presence of inhibitor was calculated using the formula below,

% Activity remaining (with inhibitor) = (slope of inhibitor sample/control slope) x 100.

## Supplementary Information

**Additional file 1: Table S1.** The statistics of clean reads generated for five different tissues (flower, leaf, seed, root, and stem) by transcriptome sequencing of MC.

**Additional file 2: Table S2.** Statistics of BUSCO analysis to assess the completeness of Moringa species transcriptome. The percentage value of completeness shown in C: Complete; S: Complete and single-copy; D: Complete and duplicated; F: Fragmented; M: Missing; N: number of genes.

**Additional file 3: Table S3.** Function annotation of unigenes identified from MC and MO transcriptome assembly. GO terms derived from homologues were classified as biological process, molecular function, and cellular component.

**Additional file 4: Fig. S1.** The top ten species distribution, functions, and domains of the MC and MO transcriptomes visualised using a pie chart. A) Species distribution of homologue hits B) Functions predicted from homologues C) Pfam domain enrichment.

**Additional file 5: Table S4.** The top ten highly expressed transcripts found in various Moringa species tissues. Transcripts with unidentified functions are referred to as "Unknown."

**Additional file 6: Table S5.** Transcription factors identified from Moringa species and closely related plants.

**Additional file 7: File S1.** Mining enzymes involved in the biosynthesis of quercetin, benzylamine, and chlorogenic acid, as well as the validation of true hits using phylogeny and FIR mapping

**Additional file 8: Table S6.** The abundance (TPM values) of transcripts encoding enzymes involved in the biosynthesis of quercetin, benzylamine, and chlorogenic acid in five different MC and MO tissues.

**Additional file 9: Fig. S2.** Quantification of the compounds quercetin (Q), chlorogenic acid (CGA), and benzylamine (BA) from crude leaf tissue extract of MC and MO using HPLC analysis (at 254 nm). The diagrams depicting A) Standard curves for the Q, CGA and BA B) HPLC trace of standards (lowest concentration 0.39 ng on column for Q, CGA and 7.81 ng on column for BA). C) HPLC trace of the standards (highest concentration 50 ng on column for Q, CGA and 1 μg on column for BA). D) HPLC trace of MC crude leaf extract. E) HPLC trace of crude leaf extract.

**Additional file 10: Table S7.** Metabolite profiling of crude leaf extract from MC and MO. A) The concentration of quercetin, chlorogenic acid, and benzylamine in MC and MO crude leaf extract by HPLC-PDA analysis. B) The compounds identified by LC-MS analysis in crude leaf extracts of MC and MO. Compound Discoverer 3.2 was used to annotate the compounds from libraries mzCloud, Mass Banks, KEGG, HMD, Chemspider, and PlantCyc.

**Additional file 11: Table S8.** Primers designed for enzymes involved in quercetin, benzylamine, and chlorogenic acid biosynthesis. These primers were used for RT-qPCR analysis.

**Additional file 12: Table S9.** The details of parameters used in HPLC PDA and LC-MS system setup.

## Data Availability

The data generated for this study can be found: National Center for Biotechnology Information (NCBI) BioProject database under accession number PRJNA665353 (https://www.ncbi.nlm.nih.gov/bioproject/PRJNA665353)

## Abbreviations

MO: *Moringa oleifera* Lam
MC: *Moringa concanensis* Nimmo
4CL: Coumaroyl CoA ligase
CHS: Chalcone synthase
CHI: Chalcone flavanone isomerase;
FLS: Flavonol synthase
F3H: Flavonol 3 hydroxylase
OMT: Tricin synthase
F3’H: Flavonoid 3 monooxygenase
NFD: N-substituted formamide deformylase
HCT: Hydroxycinnamoyl-CoA shikimate hydroxycinnamoyl-transferase
HQT: Hydroxycinnamoyl-CoA quinate hydroxycinnamoyl-transferase
C3’H: p-Coumaroyl ester 3’-hydroxylase
TPM: Transcript per million
RT-qPCR: Reverse transcription-quantitative polymerase chain reaction
HPLC-PDA: High-performance liquid chromatography–photodiode array
LC-MS: Liquid chromatography–mass spectrometry
DPP-IV: Dipeptidyl peptidase-4
GLP-1: Glucagon-like peptide 1
GIP: Gastric inhibitory polypeptide
