# Supplementary figures and images for: Transcriptome profiling of two Moringa species and insights into their antihyperglycemic activity
Source: BMC Plant Biol. 2022 Dec 2;22:561. doi: 10.1186/s12870-022-03938-6 (PMC9717441; doi:10.1186/s12870-022-03938-6)

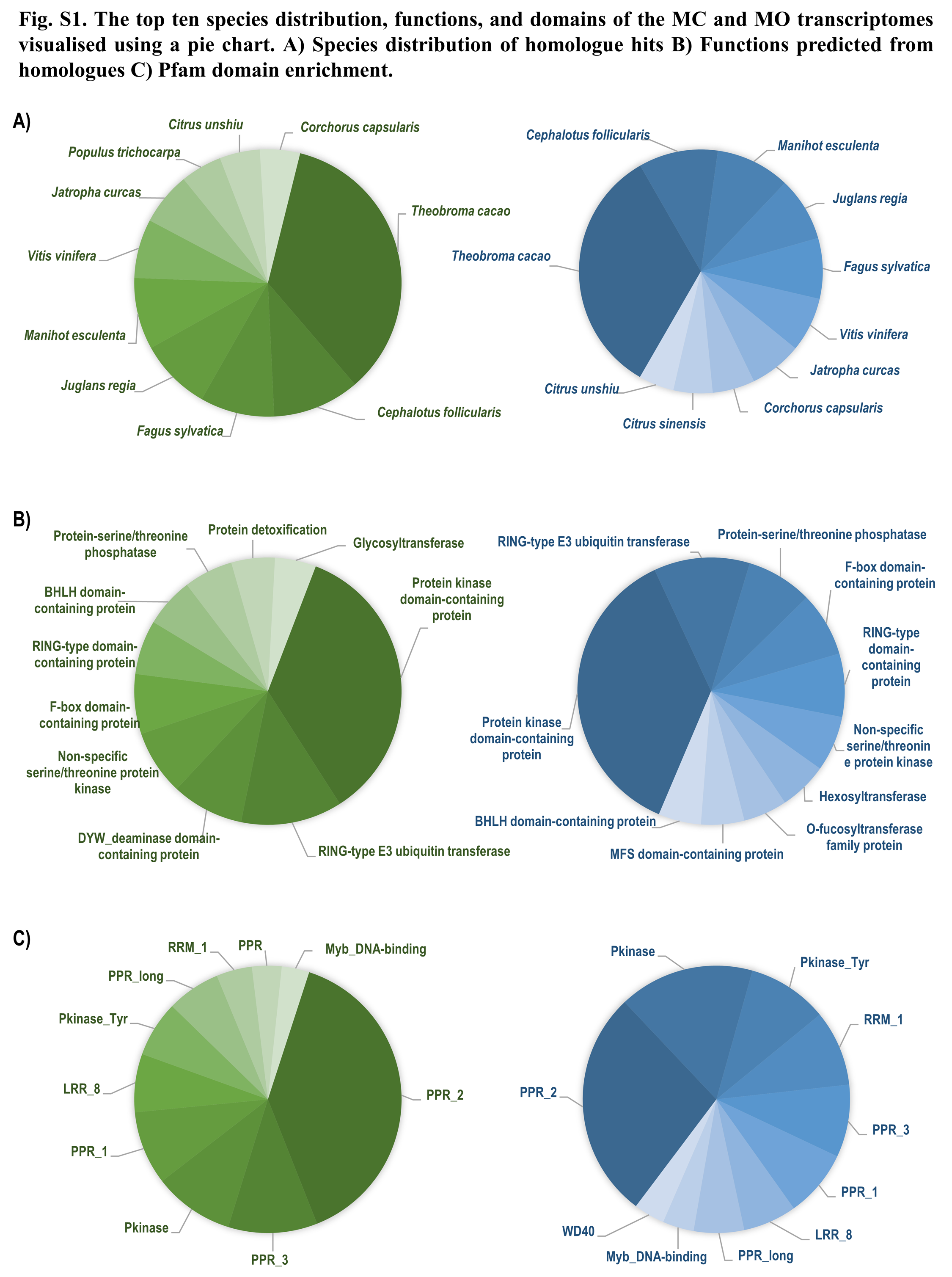

Supplement: Supplementary file 4 — Additional file 4: Fig. S1. The top ten species distribution, functions, and domains of the MC and MO transcriptomes visualised using a pie chart. A) Species distribution of homologue hits B) Functions predicted from homologues C) Pfam domain enrichment. [file 12870_2022_3938_MOESM4_ESM.tif]

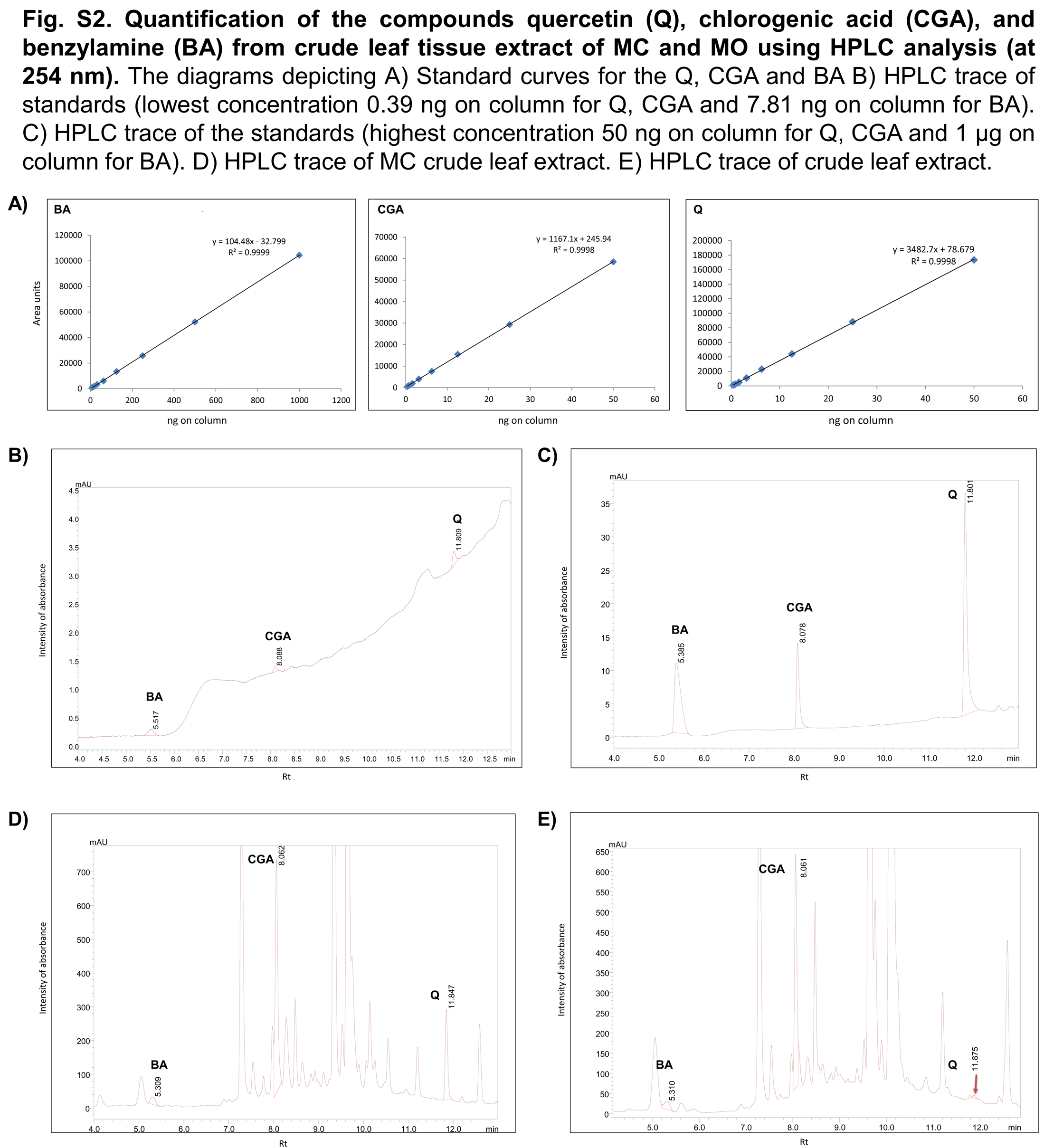

Supplement: Supplementary file 9 — Additional file 9: Fig. S2. Quantification of the compounds quercetin (Q), chlorogenic acid (CGA), and benzylamine (BA) from crude leaf tissue extract of MC and MO using HPLC analysis (at 254 nm). The diagrams depicting A) Standard curves for the Q, CGA and BA B) HPLC trace of standards (lowest concentration 0.39 ng on column for Q, CGA and 7.81 ng on column for BA). C) HPLC trace of the standards (highest concentration 50 ng on column for Q, CGA and 1 μg on column for BA). D) HPLC trace of MC crude leaf extract. E) HPLC trace of crude leaf extract. [file 12870_2022_3938_MOESM9_ESM.tif]
