# Supplementary material for: Transcriptome profiling of two Moringa species and insights into their antihyperglycemic activity
Source: BMC Plant Biol. 2022 Dec 2;22:561. doi: 10.1186/s12870-022-03938-6 (PMC9717441; doi:10.1186/s12870-022-03938-6)

File S1. Mining enzymes involved in the biosynthesis of quercetin, benzylamine, and chlorogenic acid, as well as the validation of true hits using phylogeny and FIR mapping

Quercetin biosynthesis

Pathway

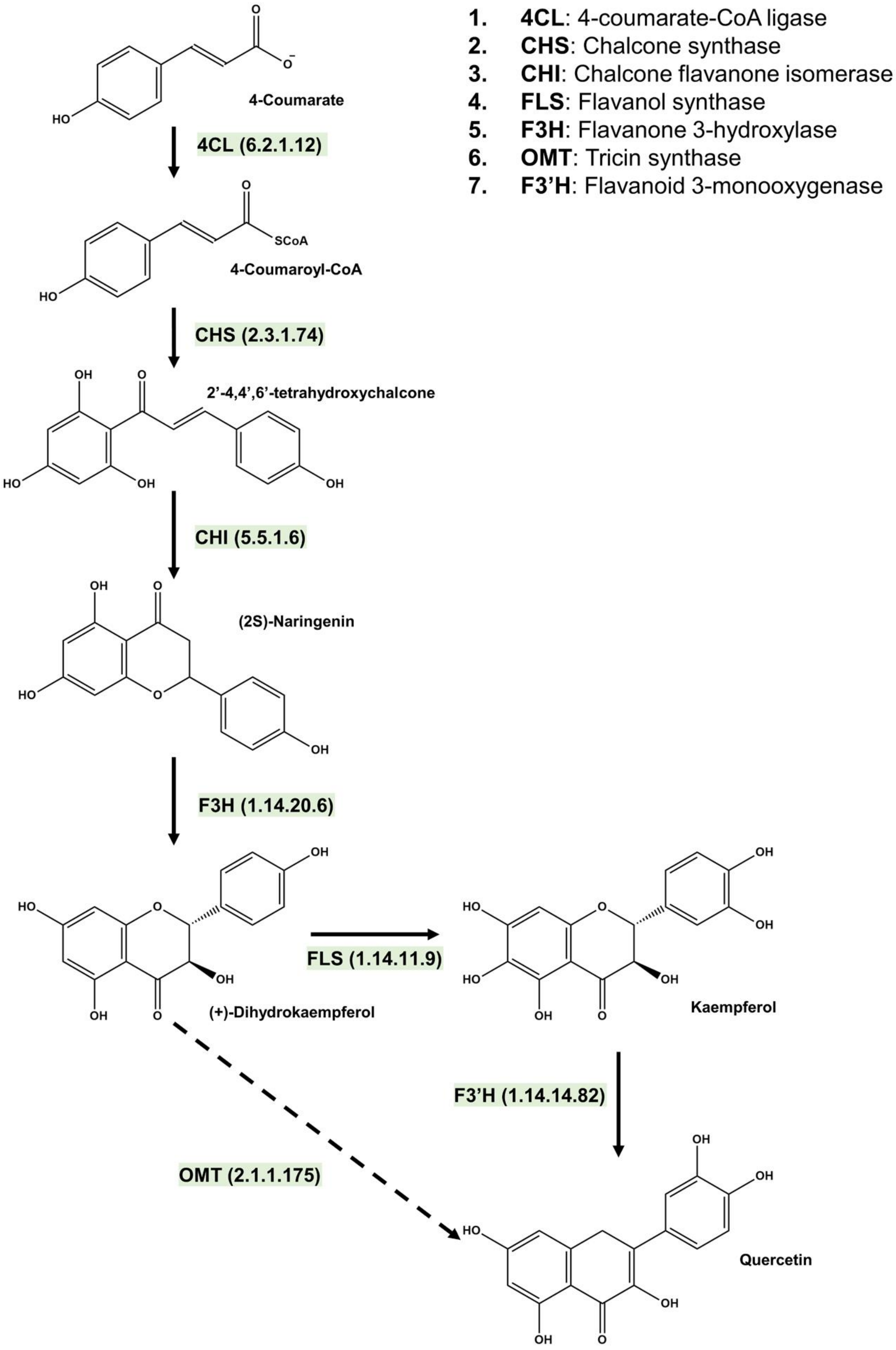

1. 4-coumarate-CoA ligase (4CL)

Phylogeny

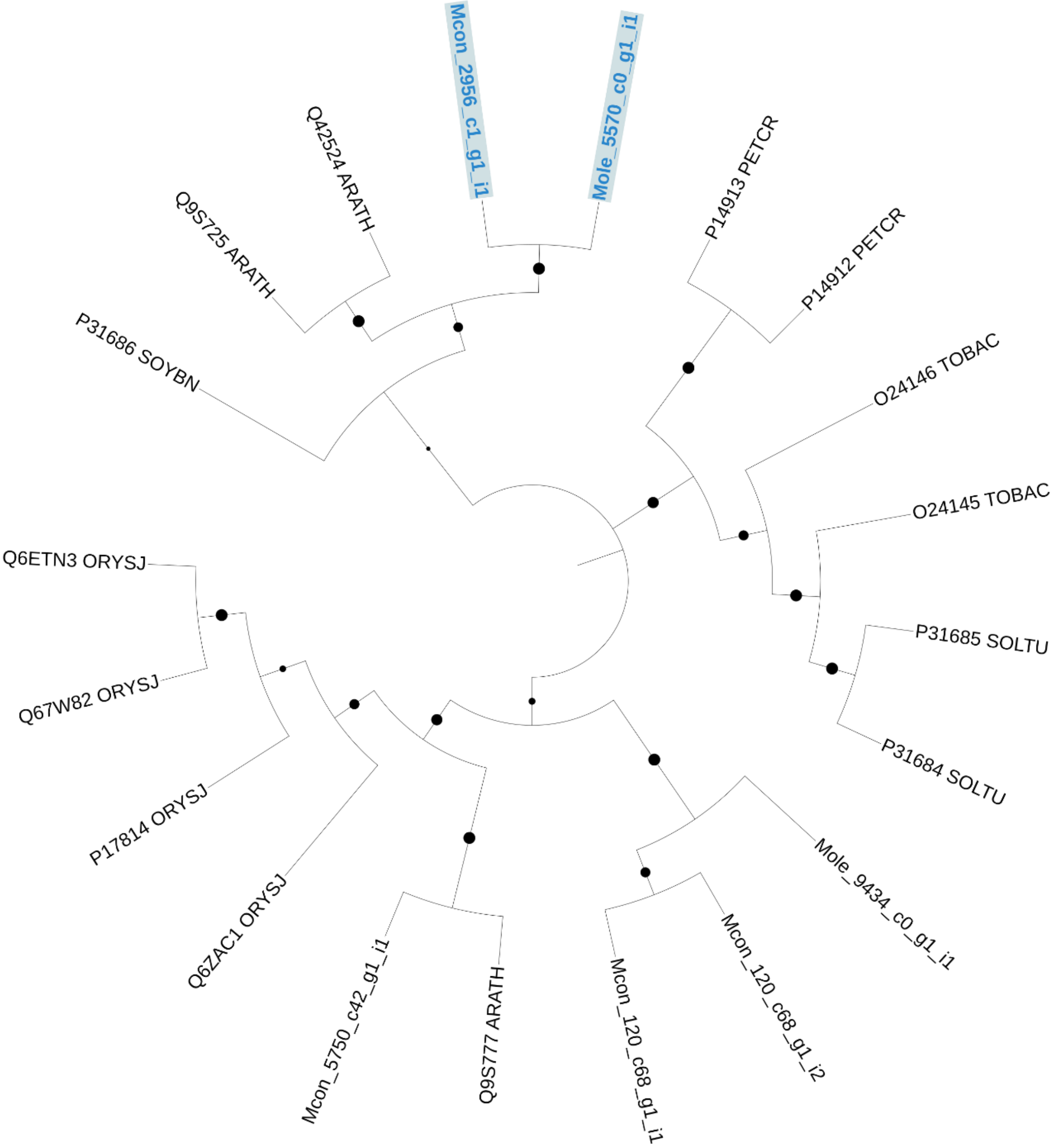

FIR mapping

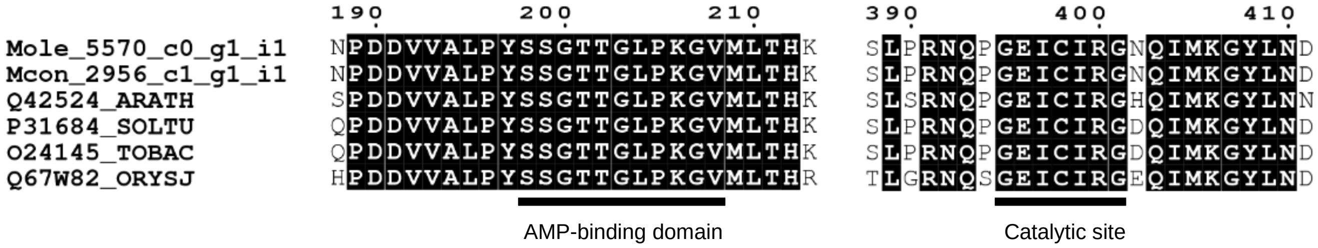

2. Chalcone synthase (CHS)

Phylogeny

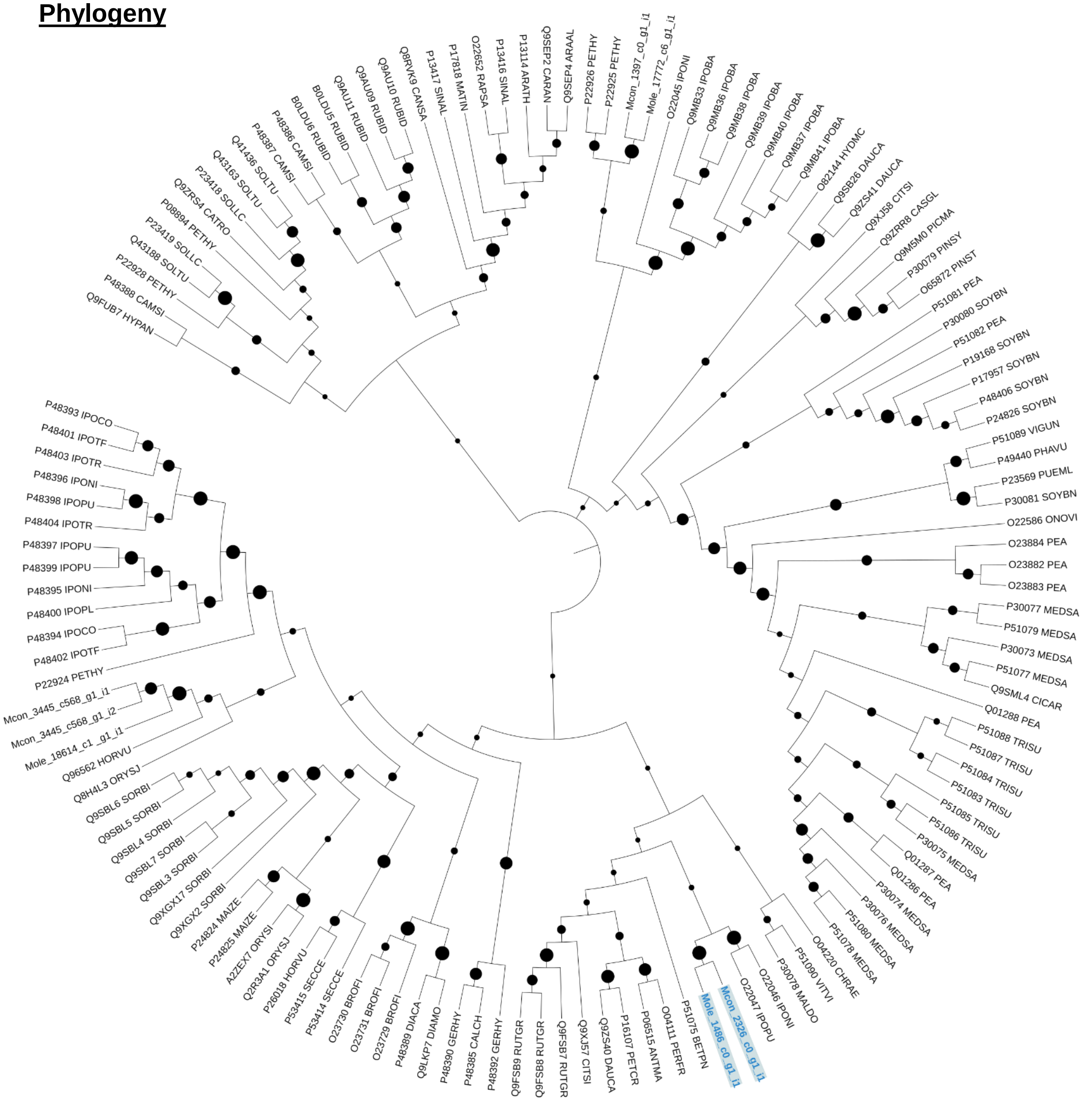

FIR mapping

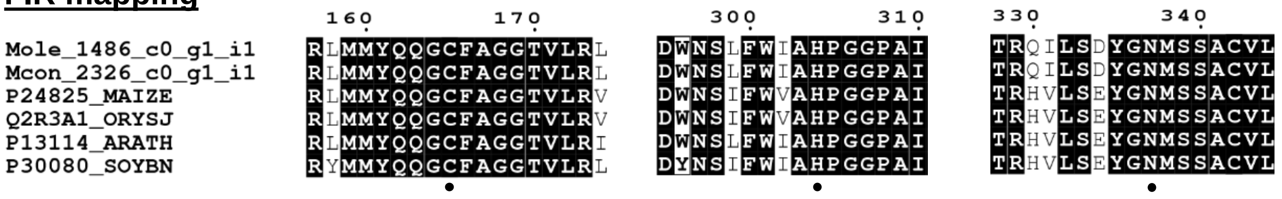

• Catalytic residues

3. Chalcone flavanone isomerase (CHI)

Phylogeny

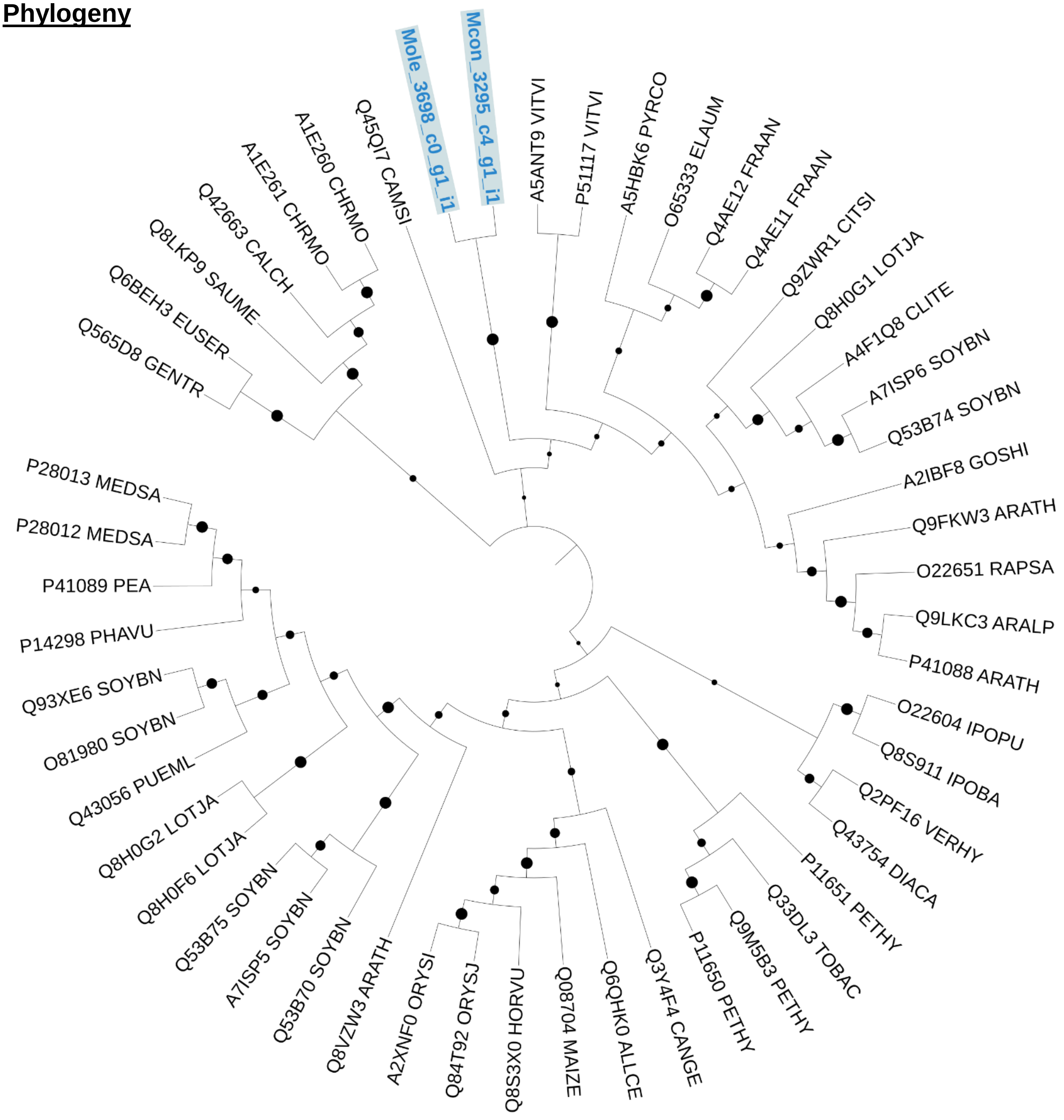

FIR mapping

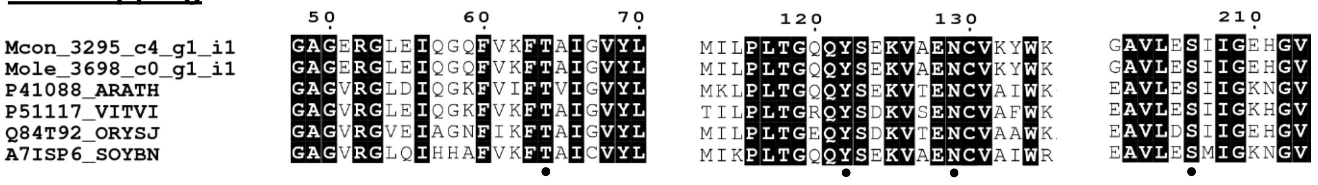

• Binding site residues

4. Flavanol synthase (FLS) / 5. Flavanone 3-hydroxylase (F3H)

Phylogeny

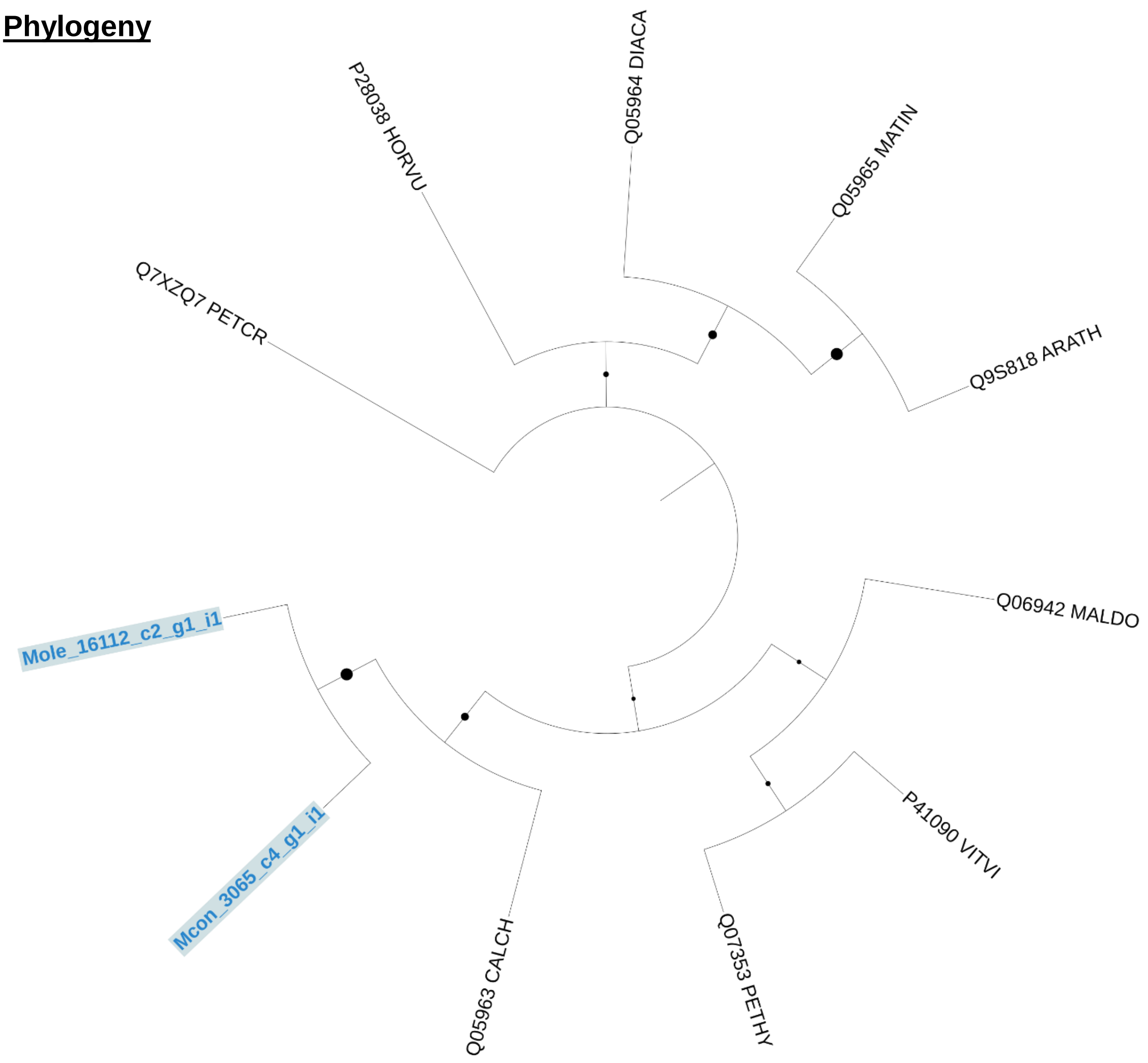

FIR mapping

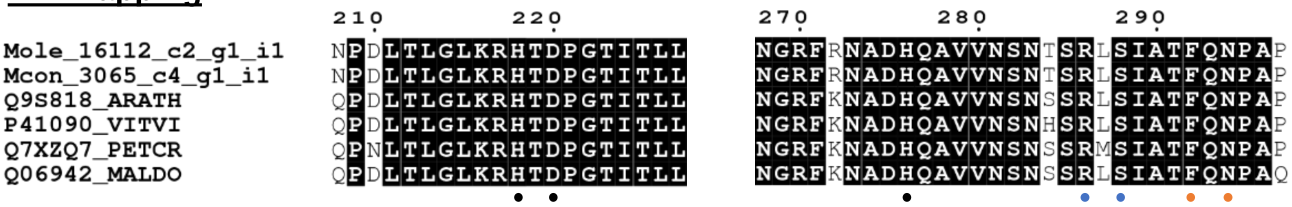

- Ferrous iron binding residues
- 2-oxoglutarate binding residues
- Substrate binding residues

5. Tricin synthase (OMT)

Phylogeny

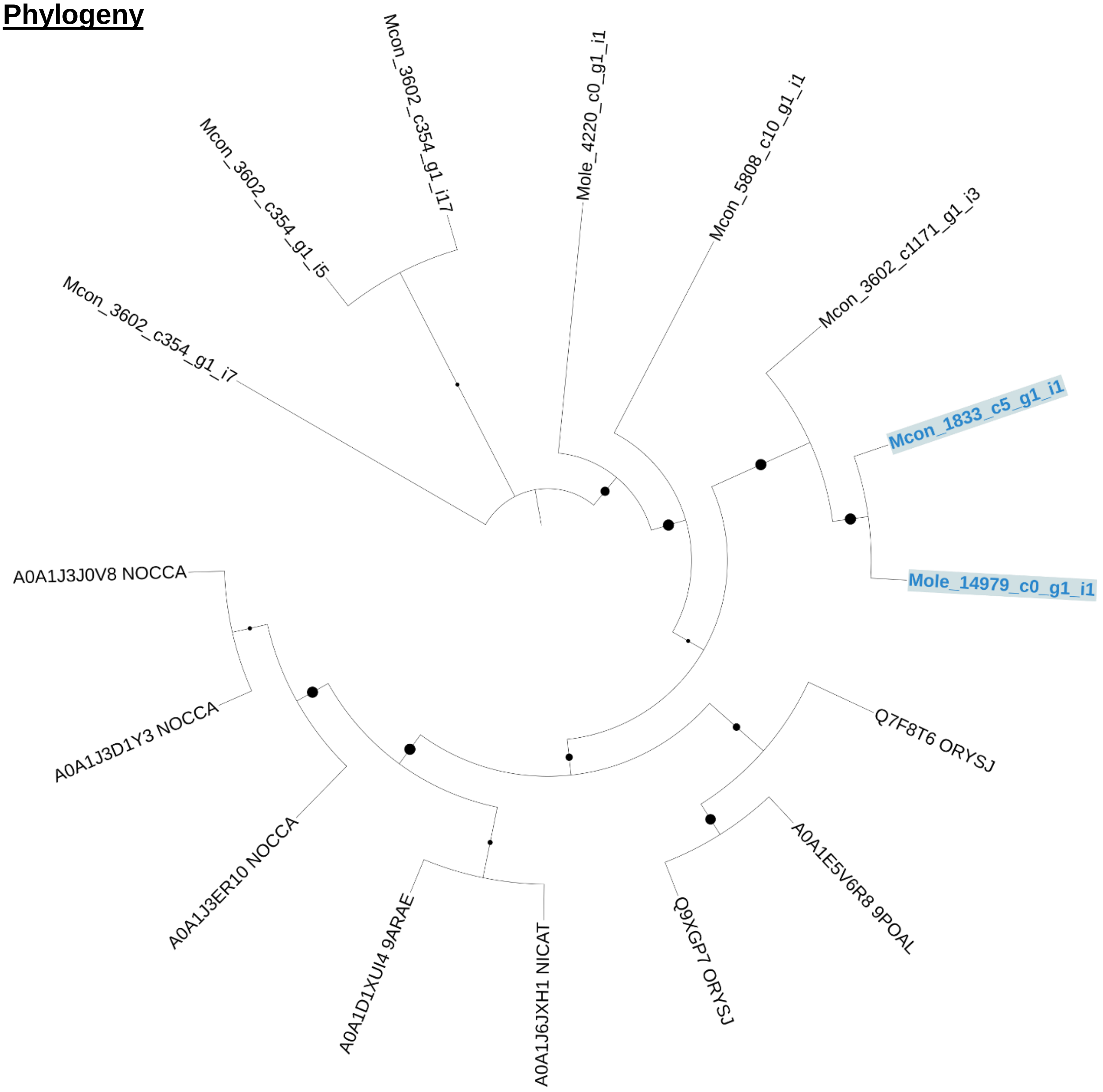

FIR mapping

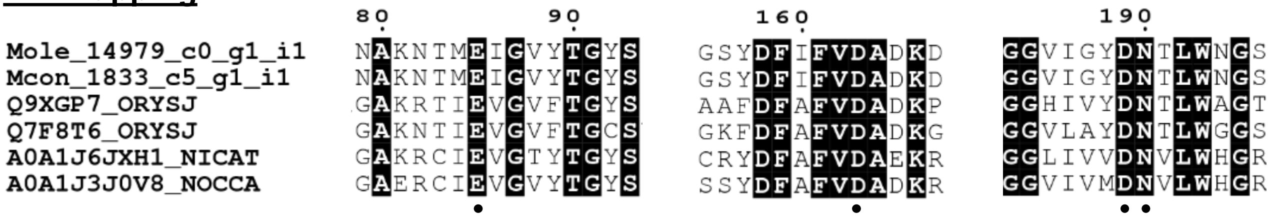

• Metal binding residues

6. Flavanoid 3-monooxygenase (F3’H)

Phylogeny

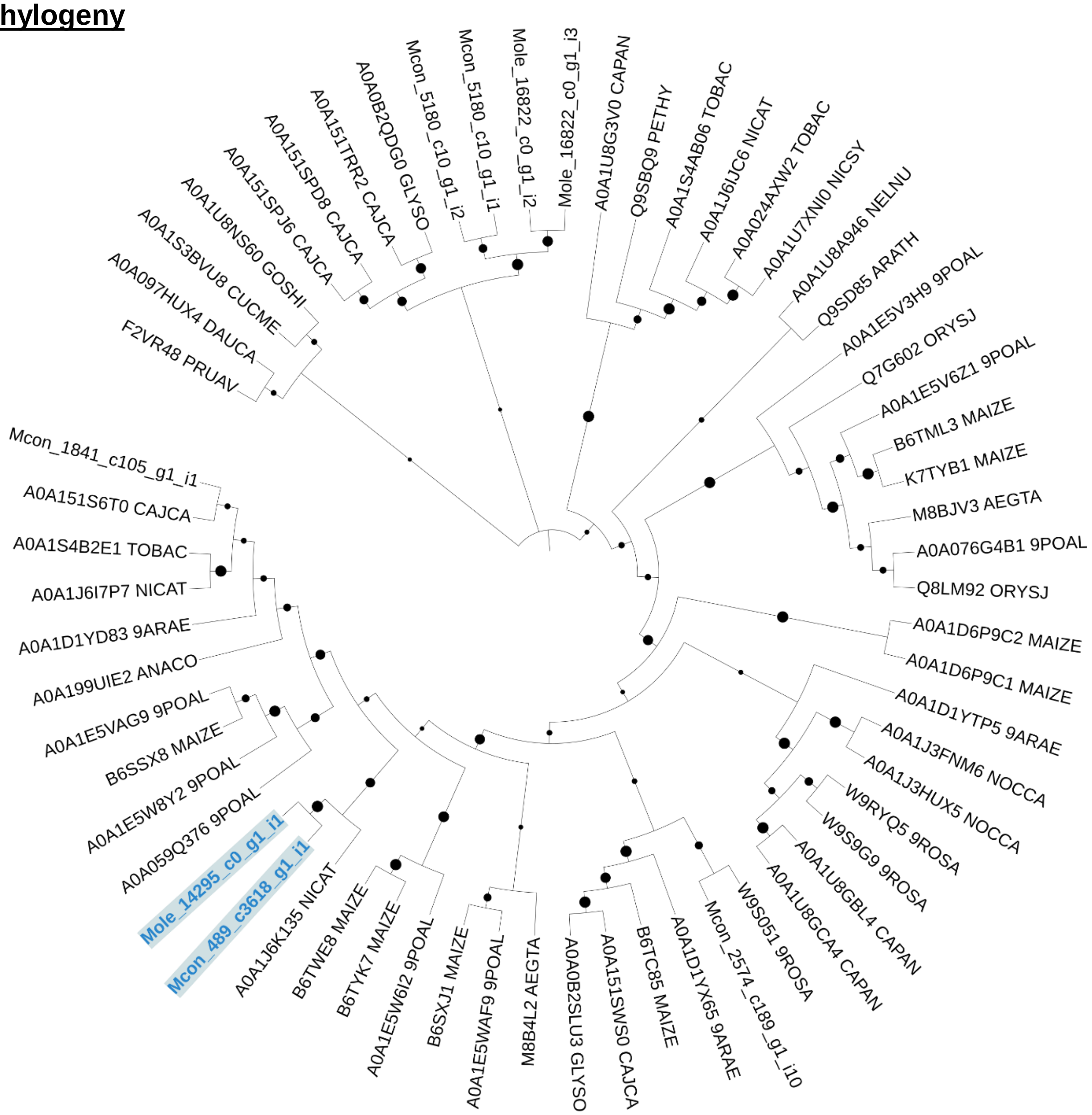

FIR mapping

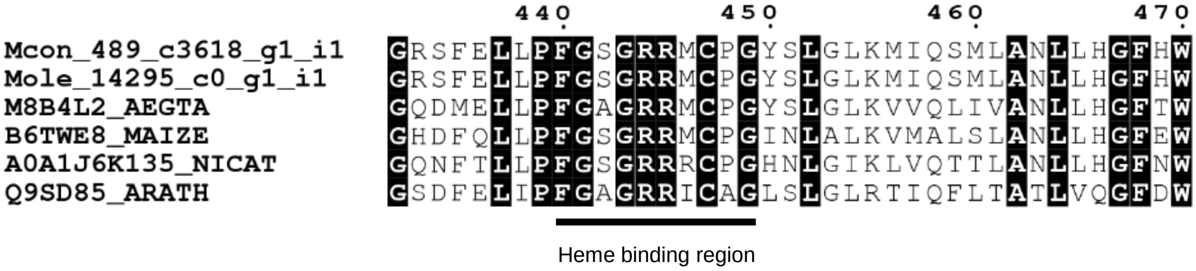

Benzylamine biosynthesis

Pathway

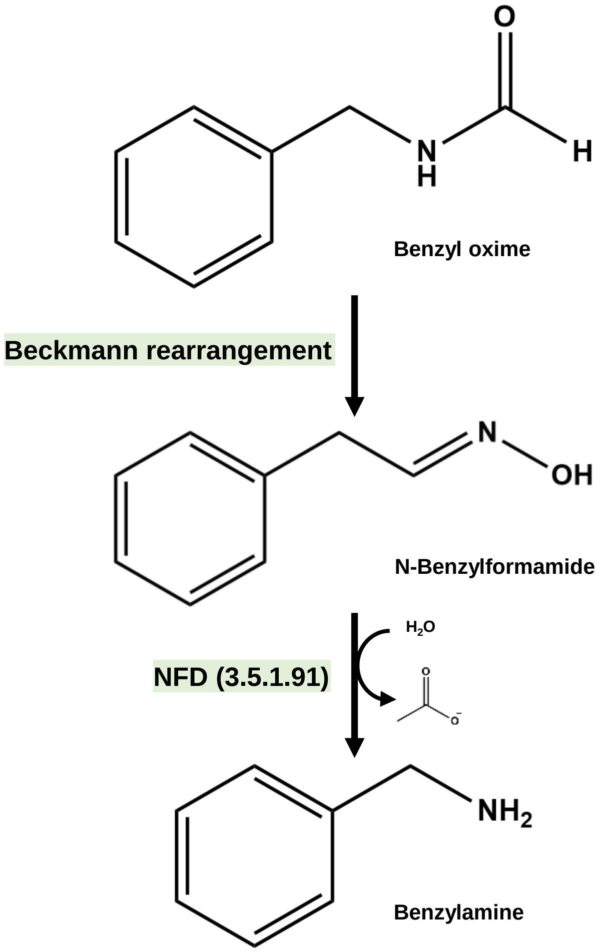

1. **NFD**: N-substituted formamide deformylase

1. N-substituted formamide deformylase (NFD)

Phylogeny

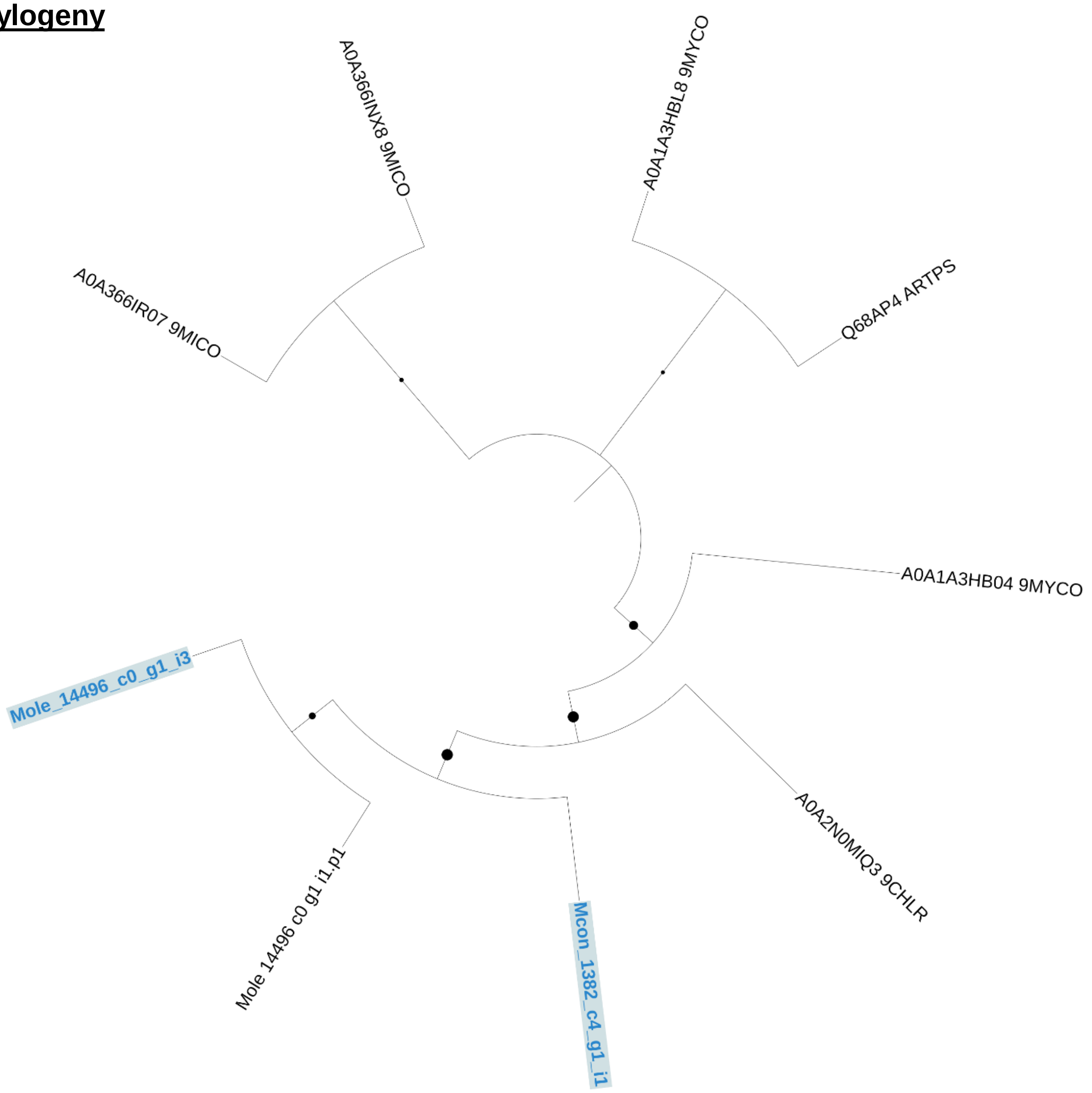

FIR mapping

	100 – 110	380	410 – 420	470 – 480
Mole_14496_c0_g1_i3	RKVVVPGFIDSHVHLIFGGL	ASDESGLQVAIH	QDRRFRIEHAQHLAPG	SRGALLALGSDWPVVDI
Mcon_1382_c4_g1_i1	RKVVVPGFIDSHVHLIFGGL	ASDESGLQVAIH	QDRRFRIEHAQHLAPG	SRGALLALGSDWPVVDI
A0A2N0MIQ3_9CHLR	GRTLLPAFSDPHNHFSINTL	QAHKRGMQVAIH	EEPRFRIEHFTITSIA	DQGLTVSGSSDYPCGPL
A0A366INX8_9MICO	GRAVVPGFIDAHNHMSIAAF	TAAAKGIDLAIH	DDPTLRLEHAFIAEKA	DAGVRVSFASDHPCGTY
Q68AP4_ARTPS	GKTVVPGFIDAHNHLSVAAF	RASKLGINLAIH	ADTVLRLEHAFIAETG	DAGVRVSLASDHPCGTY
A0A1A3HBL8_9MYCO	GRIVVPGFIDVHNHLSISAF	AAGDLGIGTAIH	IDSVLRMEHAFIAAPE	DAGVRVTLASDHPCGPF

• Metal binding residues

Chlorogenic acid biosynthesis

Pathway

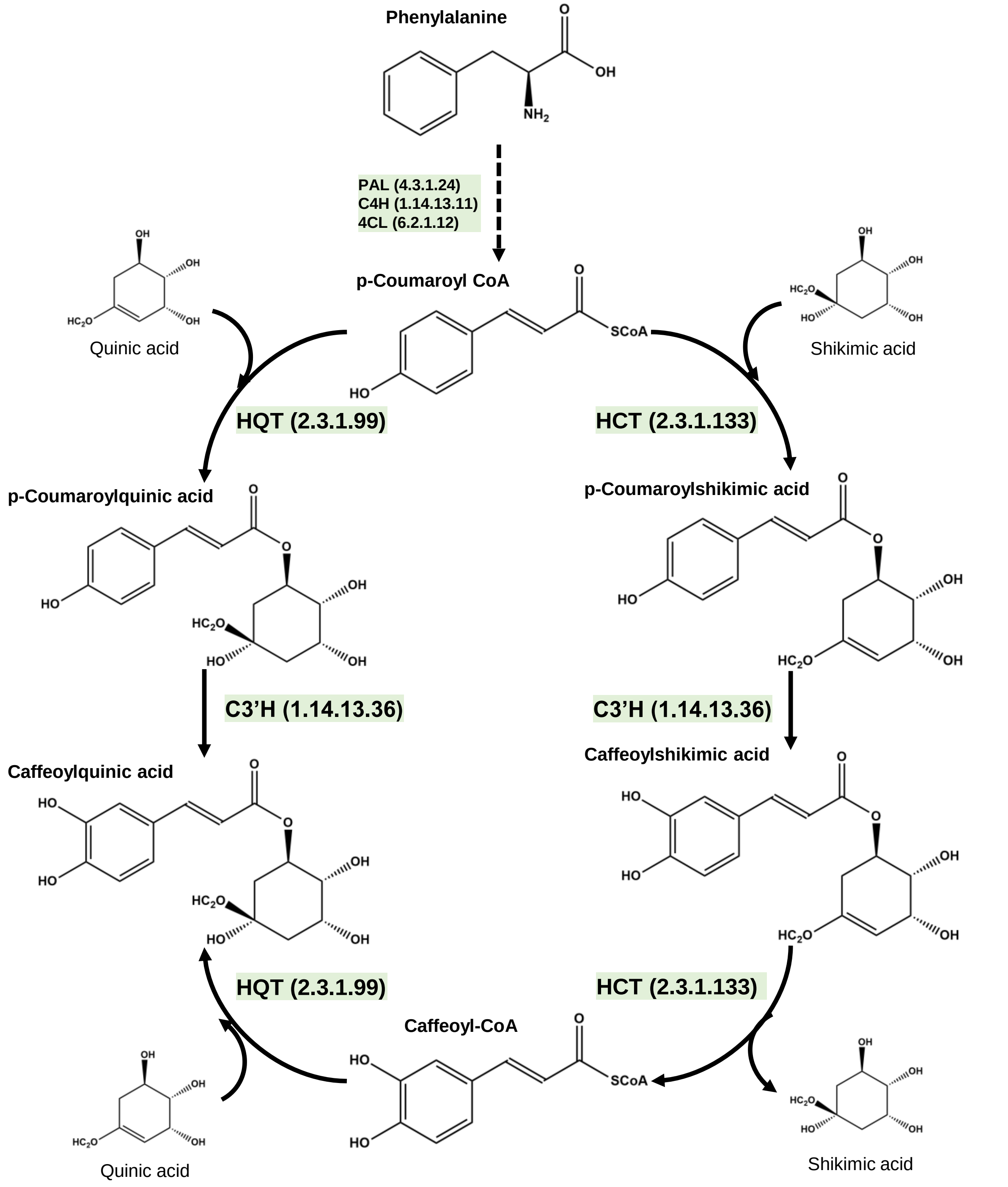

1. **HCT**: Hydroxycinnamoyl-CoA shikimate/quinate hydroxycinnamoyl transferase
2. **HQT**: Hydroxycinnamoyl-CoA quinate hydroxycinnamoyl transferase
3. **C3'H**: *p*-coumaroyl ester 3-hydroxylase

1. Hydroxycinnamoyl-CoA shikimate/quinate hydroxycinnamoyl transferase (HCT)

Phylogeny

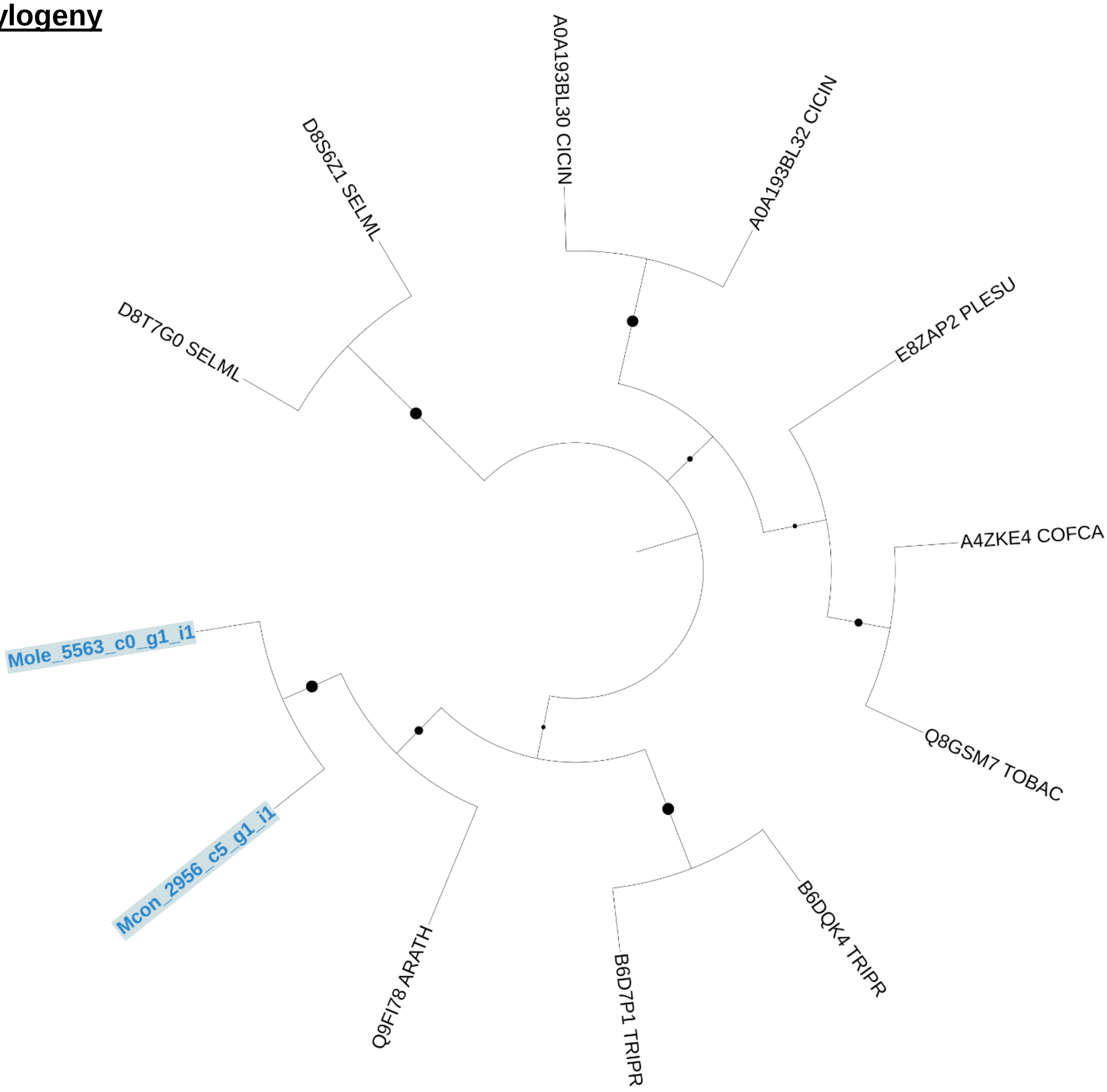

FIR mapping

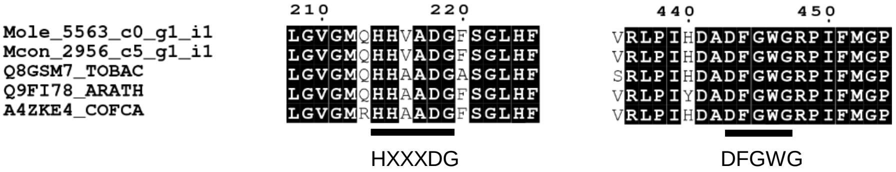

Conserved BAHD acyltransferase domains

2. Hydroxycinnamoyl-CoA quinate hydroxycinnamoyl transferase (HQT)

Phylogeny

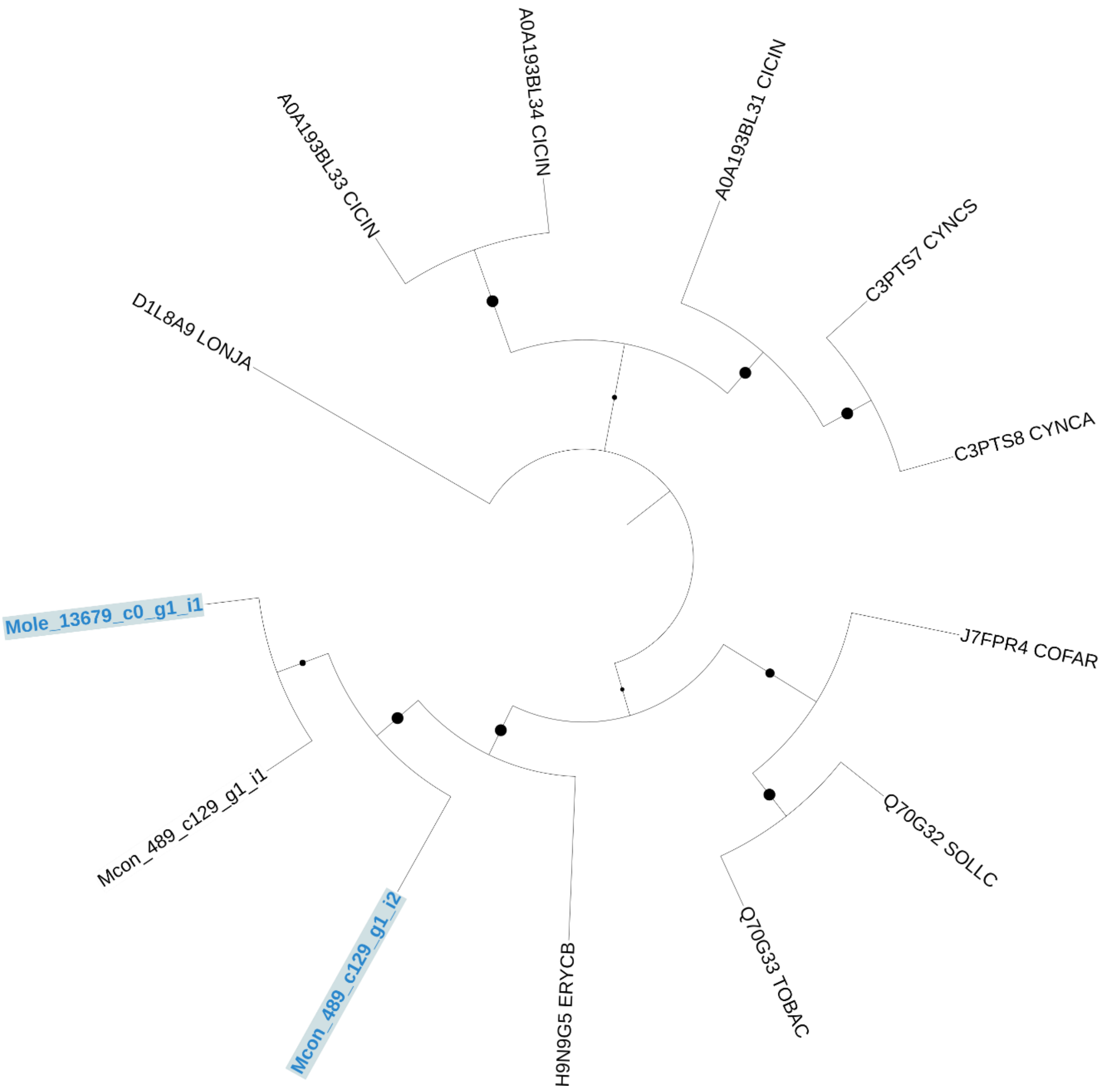

FIR mapping

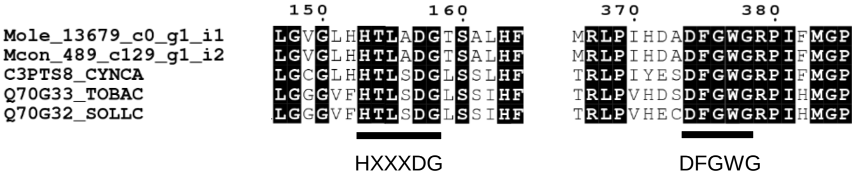

Conserved BAHD acyltransferase domains

3. *p*-coumaroyl ester 3-hydroxylase (C3’H)

Phylogeny

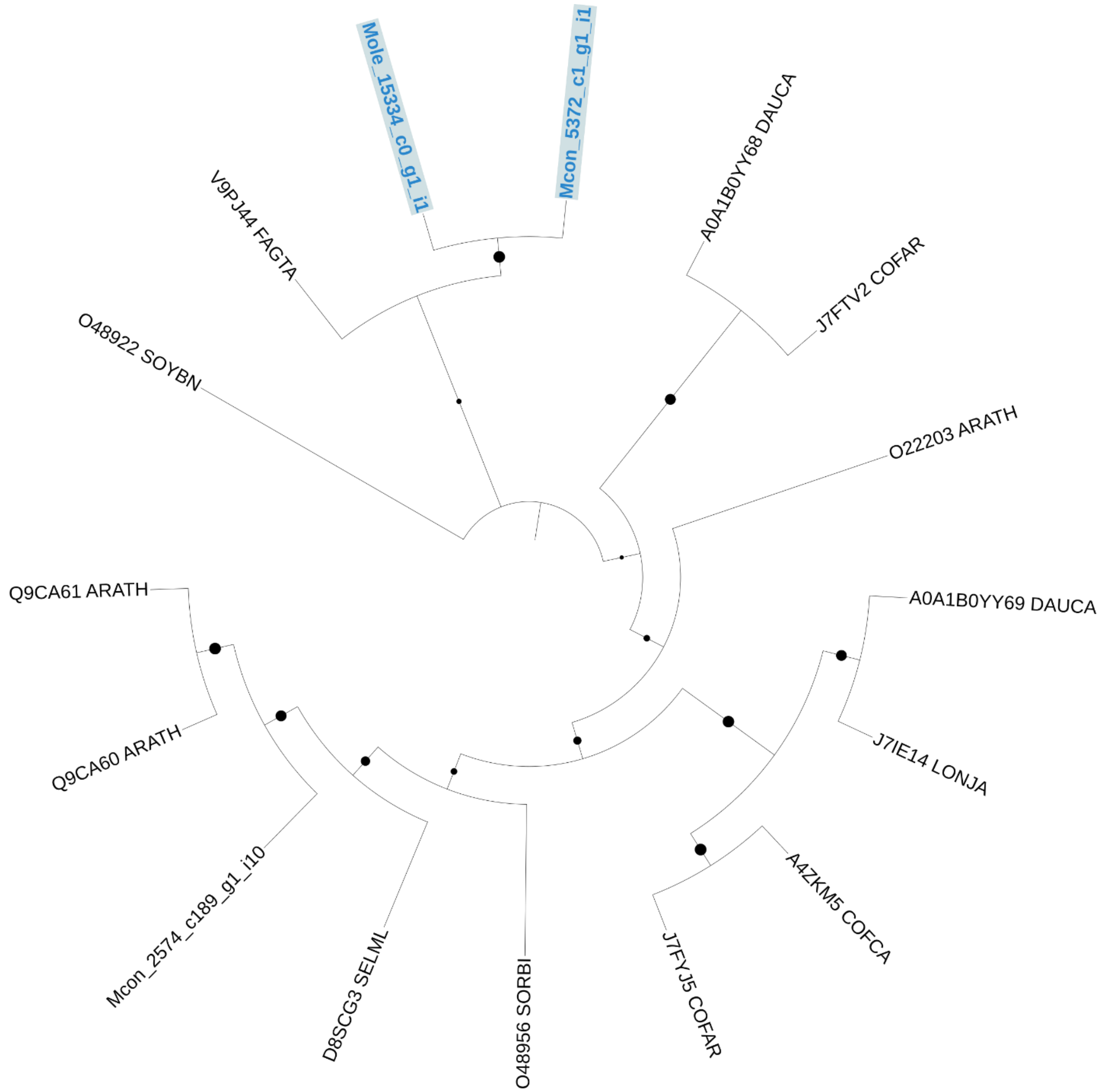

FIR mapping

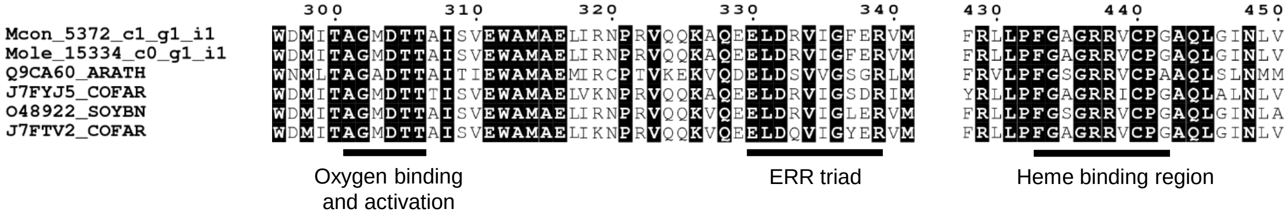

Supplement: Supplementary file 7 — Additional file 7: File S1. Mining enzymes involved in the biosynthesis of quercetin, benzylamine, and chlorogenic acid, as well as the validation of true hits using phylogeny and FIR mapping [file 12870_2022_3938_MOESM7_ESM.pdf]
